# Loss of erythrocyte sialic acid in sepsis disrupts inhibitory Siglec interactions, driving neutrophil hyperactivation and NET outspread

**DOI:** 10.1073/pnas.2536989123

**Published:** 2026-06-10

**Authors:** Anna Such, Weronika Ortmann, Gabriela Burczyk, Jacek Czepiel, Monika Bociaga-Jasik, Paweł Link-Lenczowski, Elzbieta Kolaczkowska

**Affiliations:** ^a^ Department of Experimental Hematology, Faculty of Biology, Jagiellonian University, Krakow 30-387, Poland; ^b^ Doctoral School of Exact and Natural Sciences, Jagiellonian University, Krakow 30-348, Poland; ^c^ Department of Infectious Diseases, Faculty of Medicine, Jagiellonian University Medical College, Krakow 30-688, Poland; ^d^ Department of Medical Physiology, Faculty of Health Sciences, Jagiellonian University Medical College, Krakow 31-126, Poland; ^e^ Center for the Development of Therapies for Civilization and Age-Related Diseases, Jagiellonian University Medical College, Krakow 31-066, Poland

**Keywords:** neutrophils, neutrophil extracellular traps (NETs), erythrocytes, sepsis, siglec receptors

## Abstract

Systemic inflammation is accompanied by overwhelming neutrophil activation, resulting in extensive formation of neutrophil extracellular traps (NETs). Although NETs are beneficial for pathogen entrapment, they become detrimental; therefore, their endogenous regulation is pivotal for survival. In this context, the observation that healthy individuals’ red blood cells (RBCs) can inhibit NET formation induced by lipopolysaccharide (LPS)-primed neutrophils is noteworthy. However, during systemic inflammation, NET formation in the vasculature is robust despite constant neutrophil exposure to RBCs. Herein, we reveal that for RBCs and neutrophils from septic individuals, but not from those with local inflammation, interactions cease and NET inhibition is abolished. This is observed even if only one cell type originates from an inflamed individual, and occurs in both human and murine cells. Moreover, 6 to 10 d post–sepsis induction (mice) or diagnosis (humans), during the resolution of inflammation, interactions are restored and hence fail during the critical stages of inflammation. Mechanistically, this is not due to passive LPS absorption by RBCs, which would limit its availability for neutrophils. In contrast, the cessation of NET inhibition is due to inflammation-induced RBC desialylation (removal of α2,3-linked sialic acids) and decreased expression of inhibitory Siglec-E/9 and -G, but not Siglec-F/5, on neutrophils. Accordingly, concurrent addition of exogenous polysialic acid (PolySia) and pharmacologically controlled accumulation of Siglec molecules on neutrophils preserves the ability of erythrocytes to inhibit PAD4-dependent NET formation upon interaction with neutrophils of endotoxemic mice. These findings open avenues for clinically controlling NET formation during systemic inflammation, given the abundance of erythrocytes in the blood.

## Significance

Sepsis is a life-threatening systemic inflammation with poorly understood mechanisms and no specific therapy. It is associated with overactivated neutrophils that overrelease neutrophil extracellular traps (NETs), leading to bystander cytotoxicity. One endogenous cell type that can prevent this is the erythrocyte. Red blood cells (RBCs) are the most abundant cells in the body; they are present in the blood, where sepsis originates. We report that whereas in healthy individuals (humans and mice), RBCs downregulate cytokine and NET release in response to acute activation, during sepsis, they lose this ability. We describe the causes of this change and how it can be pharmacologically reversed to presepsis neutrophil and RBC phenotypes, thereby sustaining NET inhibition during systemic inflammation and preventing adverse effects.

Neutrophil extracellular traps (NETs) play an essential role in early inflammation, trapping pathogens and limiting their dissemination. NETs are composed of chromatin fibers decorated with neutrophil proteins of granular origin, including neutrophil elastase (NE) and nuclear in origin, but citrullinated histones (1). However, persistent NET presence in the vasculature or inflamed tissues leads to bystander damage due to NET-associated histones (2) and NE-induced cytotoxicity (1). Moreover, the physiological process of NET removal can trigger new NET waves, causing further damage (3). Unlike proteases, endogenous inhibitors of NET formation have been identified only rarely to date (4). In this context, a striking observation emerges from studies of human erythrocytes (red blood cells, RBCs) from healthy individuals. These cells inhibit NET formation when incubated with neutrophils stimulated with phorbol 12-myristate 13-acetate (PMA) (5). Erythrocytes have been considered terminally differentiated cells, with functions confined to respiratory gas transport and regulation of blood rheology (6). Nonetheless, human erythrocytes have been shown to bind and scavenge inflammatory cytokines and chemokines (6), interact with nucleic acids (7) and microbial components (8), and contribute to reactive oxygen species (9) and nitric oxide generation (9).

However, in pathological conditions such as sepsis or malaria, erythrocytes can impact immune responses through hemolysis-derived danger signals (10), as some RBCs undergo hemolysis due to oxidative stress, membrane alterations, and pathogen action/invasion. Moreover, during systemic inflammation, RBCs can exhibit reduced sialic acid content on their membranes, which is associated with altered deformability and aggregability (10). Whereas it is the presence of sialic acids that otherwise helps prevent sticking to each other and the blood vessel walls. Sialic acids also form a platform for communication with leukocytes via Siglec molecules, functioning as endogenous “self” signals that suppress immune activation (11). Sialic acids constitute a family of structurally related nine-carbon monosaccharides terminating glycoconjugates in vertebrates which can modulate immune interactions (12). Humans predominantly express N-acetylneuraminic acid (Neu5Ac), whereas mice additionally synthesize N-glycolylneuraminic acid (Neu5Gc) via CMAH enzyme (12). These structural variations influence erythrocyte membrane glycosylation and can affect recognition by sialic acid-binding receptors such as Siglecs. Sialic acid-binding immunoglobulin-type lectins (Siglecs) are inhibitory receptors expressed on neutrophils and monocytes/macrophages that act as negative regulators of leukocyte migration during LPS-induced systemic inflammation (endotoxemia) (5). Aforementioned, human RBC-mediated inhibition of NET formation depends on Siglec-9, and we showed that extracellular vesicles (EVs) shed from intact erythrocytes inhibit LPS-induced NETs through Siglec-E (a murine ortholog of Siglec-9) expressed on neutrophils (13). Although humans are much more sensitive to LPS than rodents (at least by 1,000-fold), key pathways of glycan-dependent immune regulation, including sialic acid–Siglec interactions, are conserved. This supports the use of murine models to dissect Siglec-mediated control of innate immune responses, particularly by adjusting the dose or timing of stimulation (14).

We show that the Siglec-E/-9 neutrophil (PMN)-RBC interaction inhibited NET formation only in healthy individuals (mice or humans). Blocking Siglec-E endocytosis with genistein partially restored NET inhibition, and was further enhanced by exogenous polysialic acid. Hence, pharmacologically increasing RBC responsiveness may help control detrimental NET formation characteristic of sepsis beyond its onset.

## Results

### Isolation and Viability of Murine RBCs Enable Robust Modeling of RBC–Neutrophil Interactions.

**Fig. 1.**
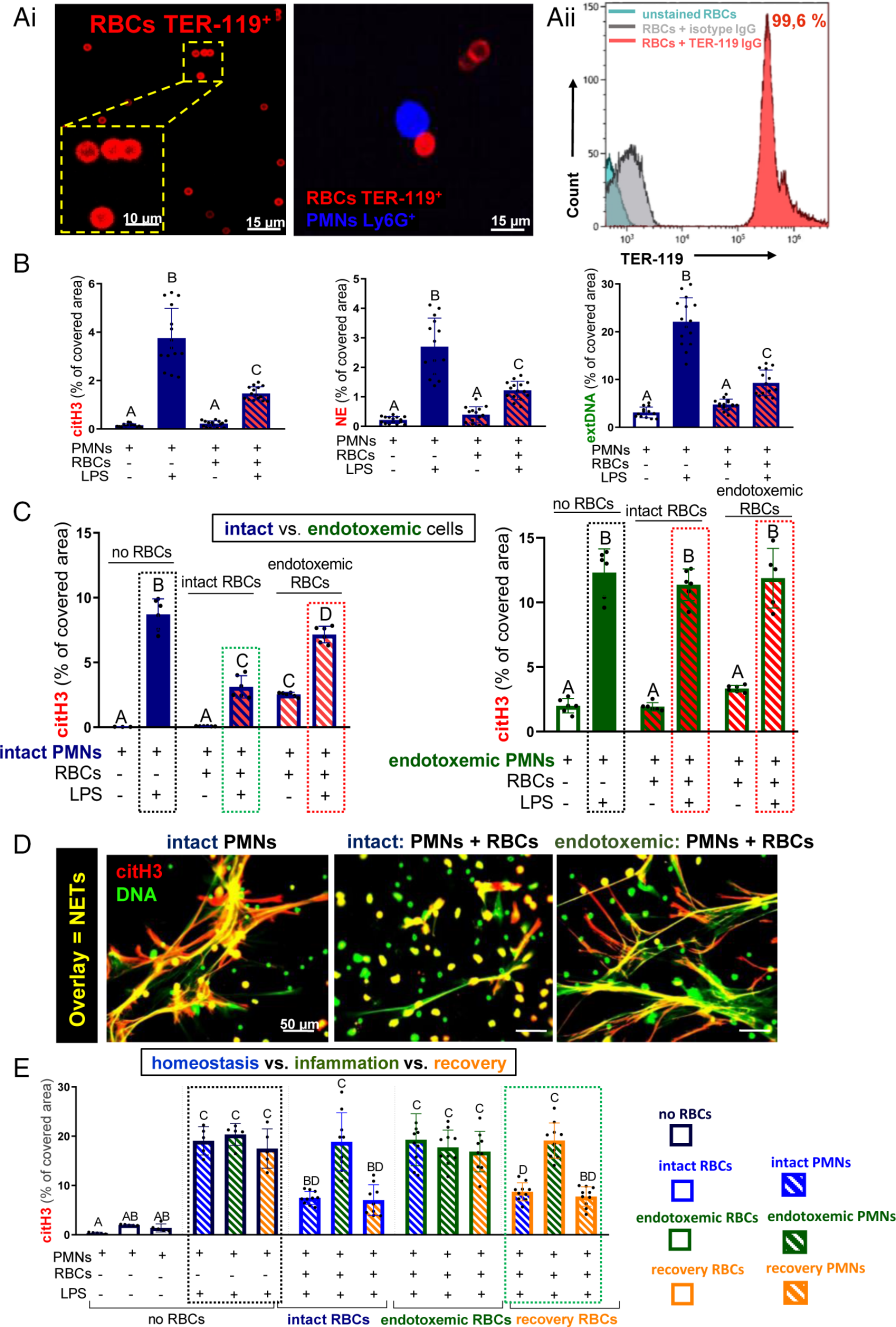
Erythrocytes of healthy mice or animals recovering from systemic inflammation, but not those with endotoxemia, inhibit NET formation. Some mice were left untreated (intact) and in those with endotoxemia some were studied at 8 h (endotoxemic cells) and some after 6 d (recovery cells). After isolation erythrocytes (RBCs) were cocultured with neutrophils (PMNs) in homo- or heterogeneous mixtures, and subsequently NET release was studied (6 h). TER-119^+^ RBC and PMN interactions (*Ai*) and expression (*Aii*). NETs from intact cells (*B*), mixed intact/endotoxemic cells (*C* and *D*), and with addition of recovery cells (*E*). In some graphs, the black frames indicate the reference group, the green frames indicate changes relative to the reference group, and the red frames indicate no change. Results are presented as mean ± SD from at least three field of view (FOV). Statistically significant differences were marked with letter symbols according to one-way ANOVA (Bonferroni post hoc); n ≥ 3; PMNs-neutrophils, RBCs-erythrocytes.

To establish an ex vivo model for studying RBCs, we developed a protocol for isolating RBCs from whole blood (Fig. 1*A*). Flow cytometry analysis using the erythroid marker TER-119 confirmed the high purity of the isolated RBCs (99.6% TER-119^+^) (Fig. 1 *Aii*). Upon coincubation with PMNs, direct cell–cell interactions were observed, suggesting their functional cross-talk (Fig. 1 *Ai*). To assess hemolysis during coculture, various cell concentrations (200 vs. 400 × 10^5^ RBCs/well) and media (HBSS −/+, PBS, RPMI 1640) were tested (*SI Appendix*, Fig. S1*A*). None significantly impacted RBC survival, confirming their stability in the coculture environment (*SI Appendix*, Fig. S1*A*). For subsequent studies, the following conditions were selected: cells cultured in HBSS+ for 6 h at 4:1 ratio (RBC:PMN).

### RBCs Enhance Neutrophil Viability and Suppress NET Formation under Acute Inflammatory Stimulation.

Building on the validated coculture system, we investigated the impact of murine RBCs on neutrophil response to inflammatory stimulation. Following 6 h of LPS challenge, PMNs displayed reduced viability, consistent with activation-induced cytotoxicity. However, coincubation with RBCs preserved neutrophil viability, whereas their mitochondrial activity remained unchanged. Furthermore, nitric oxide (NO^2−^) levels were reduced in the presence of RBCs and LPS (*SI Appendix*, Fig. S1*B*). We then examined the impact of RBCs on NET formation. At first, both cell types were isolated from untreated, healthy mice. In such a scenario, LPS-stimulated PMNs robustly released NETs, reflected by increased levels of extracellular DNA (extDNA), citrullinated histones (citH3), and NE (Fig. 1*B* and *SI Appendix*, Fig. S1 *C* and *D*). Significantly, coculture with RBCs inhibited all NET-associated markers, while RBCs alone did not induce NET formation, confirming their specific suppressive effect during LPS/bacterial activation (Fig. 1*B*). Optimization experiments confirmed that the order of cell seeding (neutrophils first or RBCs first) was not central (*SI Appendix*, Fig. S1 *C* and *D*). To determine whether the NET-suppressive effect of RBCs persists at higher PMN:RBC ratios more accurately resembling physiological conditions in mice (approximately 1:6,000), we conducted optimization experiments across an increasing range of cell ratios (1:4, 1:40, 1:400, 1:1,000). These assays revealed an apparent dose-dependent inhibition of NET formation, accompanied by a parallel increase in neutrophil viability (*SI Appendix*, Fig. S2 *A* and *B*). Notably, the regulatory effect of RBCs was consistent across LPS sources (either *Escherichia coli* or *Pseudomonas aeruginosa*) (*SI Appendix*, Fig. S2*C*).

To further test the physiological relevance of these findings and assess whether the spatial arrangement affects cell interactions, we used a 3D hydrogel-based model with Puramatrix scaffolds. In this environment, RBCs enhanced neutrophil viability following LPS stimulation, exceeding that of unstimulated controls (*SI Appendix*, Fig. S3*A*). Mitochondrial activity in PMNs cocultured with RBCs and stimulated with LPS remained unchanged; although a tendency toward increased mitochondrial activity was observed under inflammatory conditions (*SI Appendix*, Fig. S3*A*). The analysis of NET formation in the 3D system revealed that much fewer NETs (size- and density-wise) were formed in such conditions, most probably due to the matrix constraints and limited space. Nevertheless, RBCs from intact mice showed a similar tendency to inhibit NET formation, although the effect did not reach statistical significance (*SI Appendix*, Fig. S3 *B* and *C*).

### The Inhibitory Effect of RBCs on NET Formation Disappears When the Cells Are Collected from Endotoxemic Individuals.

To determine whether the immunomodulatory capacity of RBCs toward neutrophils is preserved during systemic inflammation, we modified the coculture strategy to account for the physiological origin of both cell types. Hence, neutrophils isolated from untreated (intact) mice were cocultured with RBCs from either intact or endotoxemic donors (8 h post-LPS), and NET formation was assessed following LPS stimulation. An analogical setup was applied to neutrophils derived from endotoxemic mice. Quantification of NETs revealed that RBC-mediated inhibition depended on the intact state of both cell types and was not preserved if even one originated from an endotoxemic individual (Fig. 1 *C* and *D*). Significantly, when cells were collected 6 d post–initial LPS injection (“recovery mice”), the inhibitory effect of RBCs on NET release was fully restored (Fig. 1*E*).

### Erythrocytes Do Not Sequester LPS, Hence Do Not Deprive Neutrophils of It.

**Fig. 2.**
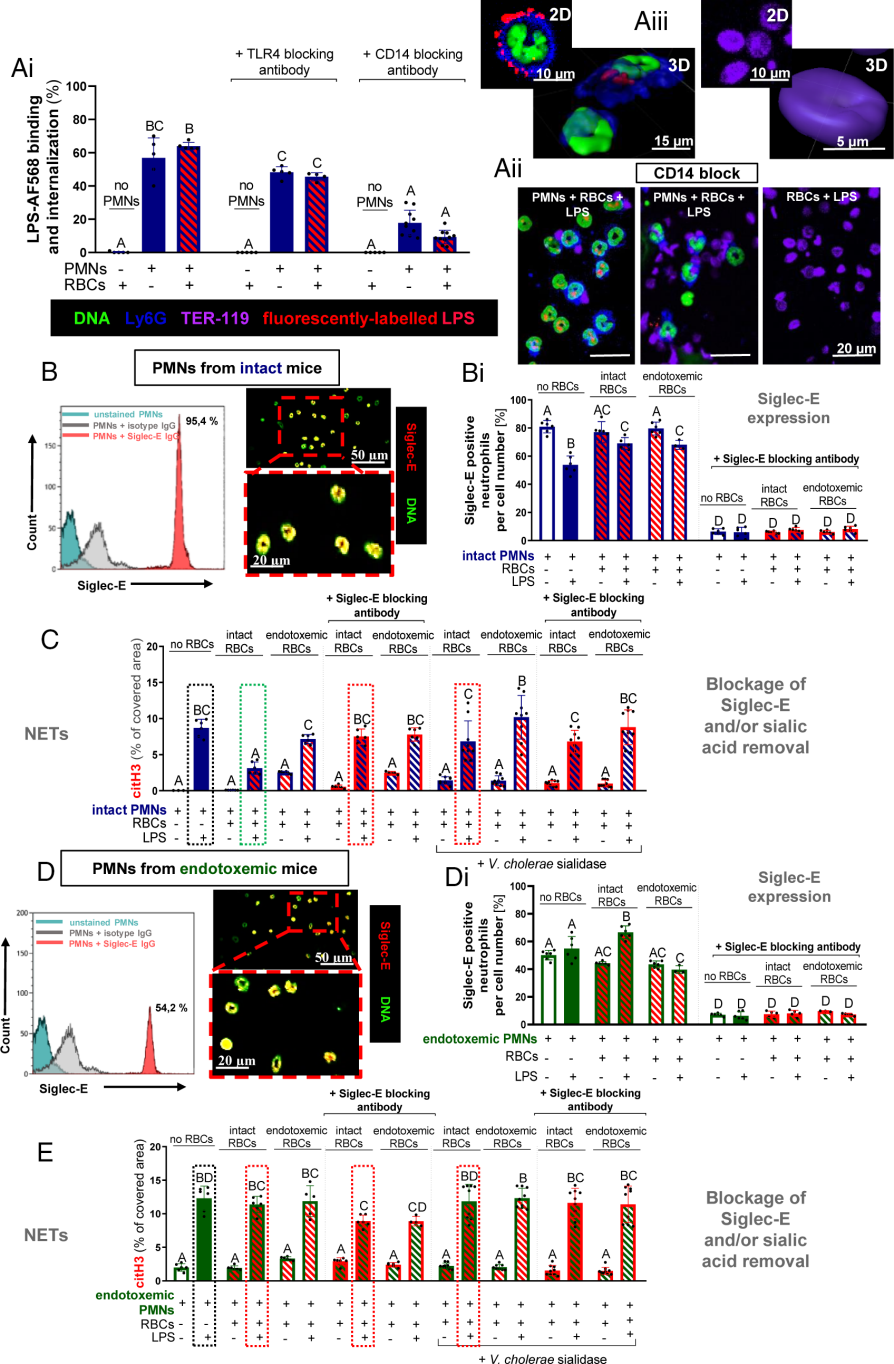
Erythrocytes do not sequester LPS, and their communication with neutrophils in mice relies on Siglec-E. Cells were isolated from intact and endotoxemic mice and cultured as mono- or cocultures of erythrocytes (RBCs) and neutrophils (PMNs), followed by fluorescent LPS stimulation. LPS binding/internalization was analyzed (*Ai*) with representative 2D (*Aii*) and 3D images (*Aiii*). Siglec-E expression (*B* and *Bi*) and NET formation (*C*) were assessed in intact PMNs, and in endotoxemic PMNs (*D* and *E*), in coculture with intact and endotoxemic RBC. In selected experiments, cells were mixed between donors, stimulated with LPS, treated with sialidase, or Siglec-E was blocked prior to analysis. Results are presented as mean ± SD from at least three FOV. Statistically significant differences were marked with letter symbols according to one-way ANOVA (Bonferroni post hoc); n ≥ 3; PMNs-neutrophils, RBCs-erythrocytes.

We then tested whether inhibition of NET formation resulted from passive LPS absorption by RBCs, as reported in other species (15). To this end, we employed fluorescently labeled LPS (LPS-AF568). Using cytospin preparations, immunofluorescent labeling, and 3D modeling (PMNs: Ly6G^+^; RBCs: TER-119^+^), we tracked LPS surface binding and internalization. No LPS uptake by erythrocytes was observed (Fig. 2 *Ai*–*Aiii*), LPS-AF568 was efficiently bound and internalized only by neutrophils (app. 65% LPS^+^ PMNs), and the intake was functionally neutralized by the CD14 blockage, rather than the TLR4 blocking antibodies, which is consistent with the literature (16). It is also significant that the presence of RBCs did not impact the LPS intake by neutrophils (Fig. 2 *Ai* and *Aii*).

To further test whether the observed effects reflected passive LPS sequestration rather than active RBC–PMN signaling, we altered the sequence of PMN stimulation. Neutrophils were either preactivated with LPS (1 h) before RBC addition, or preincubated with RBCs (1 h) before LPS exposure. In both scenarios, suppression of NET formation was preserved in cells from healthy mice (*SI Appendix*, Fig. S4 *A* and *Ai*). In contrast, RBCs derived from endotoxemic donors failed to do so (*SI Appendix*, Fig. S4*A*). Likewise, endotoxemic PMNs released high levels of NETs regardless of RBC source or sequence in which LPS was added (*SI Appendix*, Fig. S4 *B* and *Bi*).

### Siglec-E Expression and Sialic Acid-Mediated Signaling Are essential for RBC-Induced Inhibition of NET Formation.

Then we investigated the molecular basis of this selective immunoregulatory effect. Given the known role of Siglec family receptors in recognizing sialylated ligands and dampening immune responses, we initially focused on Siglec-E, an inhibitory receptor expressed on murine neutrophils. Flow cytometry and immunocytochemistry showed that the vast majority of PMNs from intact mice expressed Siglec-E (~95%), whereas this expression was significantly downregulated in endotoxemic neutrophils (~54%) (Fig. 2 *B* and *Bi* vs. Fig. 2 *D* and *Di*). Moreover, Siglec-E expression on intact PMNs declined upon LPS stimulation (Fig. 2 *Bi*, *Right*) but was partially restored by coincubation with RBCs, regardless of their origin (Fig. 2*Bi*). Notably, RBCs alone did not alter Siglec-E expression. In contrast, endotoxemic PMNs exhibited persistently lower Siglec-E levels upon LPS stimulation, unaffected by RBCs (Fig. 2 *Di*, *Right*). Interestingly, when intact RBCs were added to LPS-stimulated endotoxemic PMNs, Siglec-E expression was partially restored. In contrast, exposure to endotoxemic RBCs resulted in a pronounced additional decrease in receptor expression (Fig. 2*Di*). To functionally validate this phenomenon, we employed a blocking antibody to inhibit Siglec-E engagement. The antibody kept Siglec-E expression at low levels across all tested conditions, confirming effective receptor blockade (Fig. 2 *Bi* and *Di*).

Functionally, Siglec-E blockade altered neutrophil responses: in PMNs from healthy donors, coincubation with intact RBCs no longer suppressed NET formation (Fig. 2*C*). In endotoxemic PMNs the Siglec-E blockade did not induce any changes (Fig. 2*E* and *SI Appendix*, Fig. S4). Siglec-E functions by binding to sialic acid ligands, which induces phosphorylation of its intracellular ITIM domains and subsequently recruits the tyrosine phosphatases SHP-1 (Src homology domain 2-containing tyrosine phosphatase-1) and/or SHP-2. Indeed, we could observe recruitment of SHP-1 to Siglec-E, but only in neutrophils from intact, not endotoxemic mice (*SI Appendix*, Fig. S5).

Then we enzymatically removed surface sialic acid residues using *Vibrio cholerae* sialidase (*V. cholerae* sialidase). Remarkably, sialidase-treated intact RBCs failed to inhibit NET formation in healthy PMNs, even without Siglec-E blockade (Fig. 2*C*). Moreover, the already marginal effect of endotoxemic RBCs on NETs was further diminished by *V. cholerae* sialidase treatment. In endotoxemic PMNs, neither intact nor desialylated RBCs (from either donor group) altered NET formation, regardless of Siglec-E engagement (Fig. 2*E*). As *V*. *cholerae* sialidase has a preference for the α2,3-linked sialic acid residues, we also used a broad range Neuraminidase A (hydrolyzing α2-3, α2-6, α2-8, and α2-9 linkages), in both mouse and human systems (*SI Appendix*, Fig. S6 *A*–*Ci*). To further confirm that the linkages were digested by the enzymes we used, lectins recognizing α2-3 (MAL II) or α2-6 (SNA) residues (*SI Appendix*, Fig. S6 *D* and *Di*). These studies further documented that α2-3 desialylation is the major change in endotoxemic erythrocytes.

### Not Only NET but Also Cytokine Release Is Controlled by Siglec-E, but This Is Lost During Systemic Inflammation.

We then confirmed that RBCs also influence neutrophil effector functions beyond NETs. In intact PMNs, coincubation with intact RBCs elicited only a modest increase in IL-1β and IL-6 levels after LPS stimulation (*SI Appendix*, Fig. S7*A*). However, Siglec-E inhibition significantly increased the cytokine levels, indicating that this receptor restrains the pro-inflammatory signaling beyond NETs (*SI Appendix*, Fig. S7*A*). In contrast, intact PMNs exposed to endotoxemic RBCs showed elevated cytokine levels even without receptor blockade, with further increase upon Siglec-E inhibition (*SI Appendix*, Fig. S7*A*). A similar pattern was observed in endotoxemic PMNs (*SI Appendix*, Fig. S7*B*).

### NET Release Regulated by RBCs Is PAD4-Dependent.

To examine whether suppression of NET release by RBCs requires PAD4 activity, we utilized PAD4 knockout (PAD4^−/−^) mice as a source of PMNs and RBCs. As expected, PMNs from PAD4^−/−^ mice failed to generate NETs after LPS stimulation (*SI Appendix*, Fig. S8 *A* and *B*). In contrast, WT neutrophils cocultured with PAD4^−/−^ RBCs retained the capacity to form NETs upon LPS challenge, and NET formation remained suppressed in the presence of intact RBCs. This effect was abolished upon blocking Siglec-E, confirming that RBC-mediated regulation relies on the Siglec-E axis and that NET formation is a PAD4-dependent process (*SI Appendix*, Fig. S8).

### Siglec-9-Dependent Modulation of NET Formation by Intact RBCs Is Conserved in Human Intact Neutrophils.

**Fig. 3.**
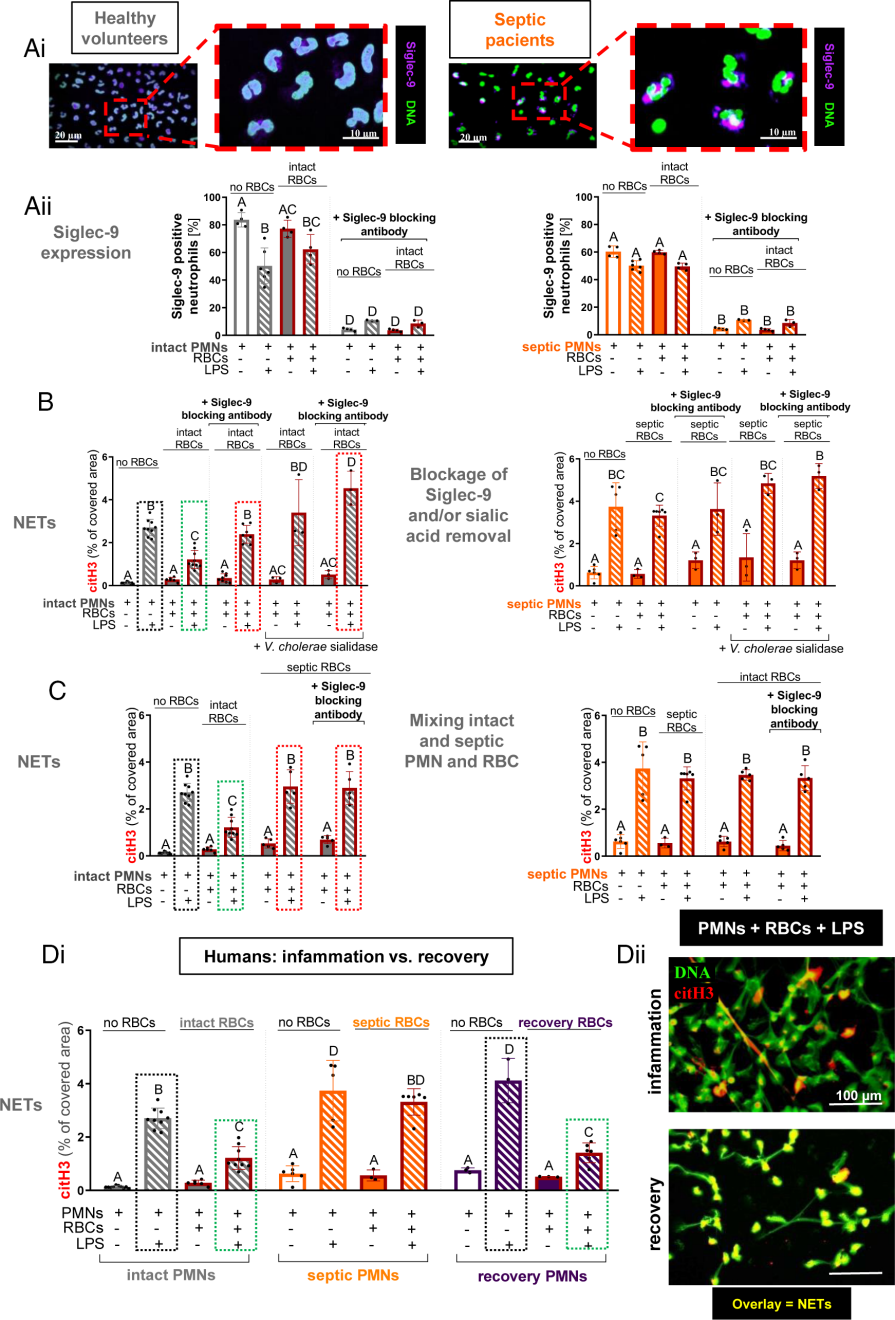
Human erythrocytes and neutrophils communicate via Siglec-9, and this interaction is disrupted when either cell type originates from septic patients. Cells were isolated from healthy donors (intact), septic patients (septic), and the same patients during recovery, and cocultured with or without LPS. In some experiments, cells from different donors were mixed. Siglec-9 expression and its blockade were analyzed (*Ai*—images, *Aii*—quantification). NET formation (citH3) was assessed under Siglec-9 blockade and after sialic acid removal (*B*), in mixed cell combinations (*C*), and in cells from septic and recovery patients compared with intact controls (*Di*—quantification, *Dii*—images). Results are presented as mean ± SD from at least three FOV. Statistically significant differences were marked with letter symbols according to one-way ANOVA (Bonferroni post hoc); n ≥ 3; PMNs-neutrophils, RBCs-erythrocytes.

Then we compared neutrophils isolated from healthy volunteers and septic patients. All clinical data/blood parameters of involved individuals are presented in *SI Appendix*, Table S1. Siglec-9 is the closest functional homolog of murine Siglec-E (5). Approximately 80% of neutrophils from healthy donors expressed Siglec-9, but only 60% of septic patient PMNs did so (Fig. 3 *Ai* and *Aii*). To evaluate whether this expression is dynamically modulated, neutrophils from healthy donors were stimulated ex vivo with LPS, RBCs, or both. LPS alone diminished Siglec-9 levels, while RBCs had no appreciable effect (Fig. 3 *Aii*, *Left*). However, coincubation with both LPS and RBCs partially preserved Siglec-9 expression. In contrast, neutrophils from septic patients exhibited uniformly low Siglec-9 levels across all treatment conditions, suggesting receptor exhaustion or impaired inducibility in systemic inflammation (Fig. 3 *Aii*, *Right*). Furthermore, RBCs from healthy donors suppressed NET release in LPS-stimulated neutrophils (Fig. 3 *B*, *Left*). In contrast, neutrophils from septic patients released abundant NETs, and this response remained unaltered in the presence of autologous RBCs (Fig. 3 *B*, *Right*). Blocking Siglec-9 signaling or removing sialic acids from RBCs with *V. cholerae* sialidase abolished this effect in healthy cells (Fig. 3*B*, *Left*). Notably, simultaneous blockade of Siglec-9 and sialidase treatment further enhanced NET release (Fig. 3 *B*, *Left*). In contrast, neutrophils from septic patients remained unresponsive to these manipulations (Fig. 3 *B*, *Right*).

### In Sepsis, RBC–Neutrophil Interactions Are Disrupted but Are Restored as Inflammation Resolves.

Consistent with murine data, NET inhibition by RBCs was observed only when both human neutrophils and RBCs were derived from healthy donors, but not those with sepsis (Fig. 3 *C*, *Left*). We included an additional group of patients with Lyme disease, a condition characterized by localized inflammation. In these individuals, both RBCs and neutrophils retained full suppressive responsiveness: RBCs inhibited NET formation in a Siglec-9-dependent and sialic acid-dependent manner, reversed by receptor blockade or *V. cholerae* sialidase (*SI Appendix*, Fig. S9 *A*–*C*). These findings suggest that the RBC–neutrophil regulatory axis remains intact during mild or localized inflammation but is selectively compromised during systemic inflammation. Hematological profiles also revealed distinct systemic changes in sepsis: increased WBC and granulocyte counts, and reduced RBC counts, hematocrit (HCT), and hemoglobin (HGB) levels (*SI Appendix*, Fig. S9*D*).

Importantly, we observed that the immunomodulatory function of RBCs recovered during the resolution of systemic inflammation (Fig. 3 *Di* and *Dii*). RBCs collected during the early phase of inflammation (days 1 to 2 after diagnosis/hospitalization) failed to inhibit NET release. However, RBCs obtained from the same patients at later time points (days 10 to 14) exhibited restored suppressive capacity (Fig. 3 *Di* and *Dii*). This functional recovery paralleled the normalization of hematological indices (*SI Appendix*, Fig. S9*E*).

### Sialylation of Erythrocyte Glycoproteins Changes Dramatically during Systemic Inflammation but Restores during Resolution.

To assess changes in N-glycosylation, structural analysis of erythrocyte-derived oligosaccharides from healthy, endotoxemic, and recovery mice was performed using MALDI-TOF MS (*SI Appendix*, Fig. S10 *A* and *B*). Some of the possible isoforms of N-linked glycans are shown in representative mass spectra (*SI Appendix*, Fig. S10*B*). N-glycans, as well as derived glycan traits (sialylation), were analyzed. Oligomannose, hybrid, and complex N-glycan structures were identified in all analyzed samples. In intact RBCs, the majority of hybrid and complex glycans were α2,3-sialylated and fucosylated (*SI Appendix*, Fig. S10*B* and Dataset S1). There was a dramatic shift in the distribution and quantity of sialic acid residues in individuals with systemic inflammation, which was fully restored as inflammation resolved. Specificity of the detection was confirmed by pretreating the sample with *V. cholerae* sialidase, which nearly completely removed sialic acid (*SI Appendix*, Fig. S10*A*). Notably, the amount of sialic acid on endotoxemic RBCs was as low as that observed upon addition of exogenous *V. cholerae* sialidase (*SI Appendix*, Fig. S10*A*).

### Exogenous Polysialic Acids Reverse the Effect of Desialylated Erythrocytes.

**Fig. 4.**
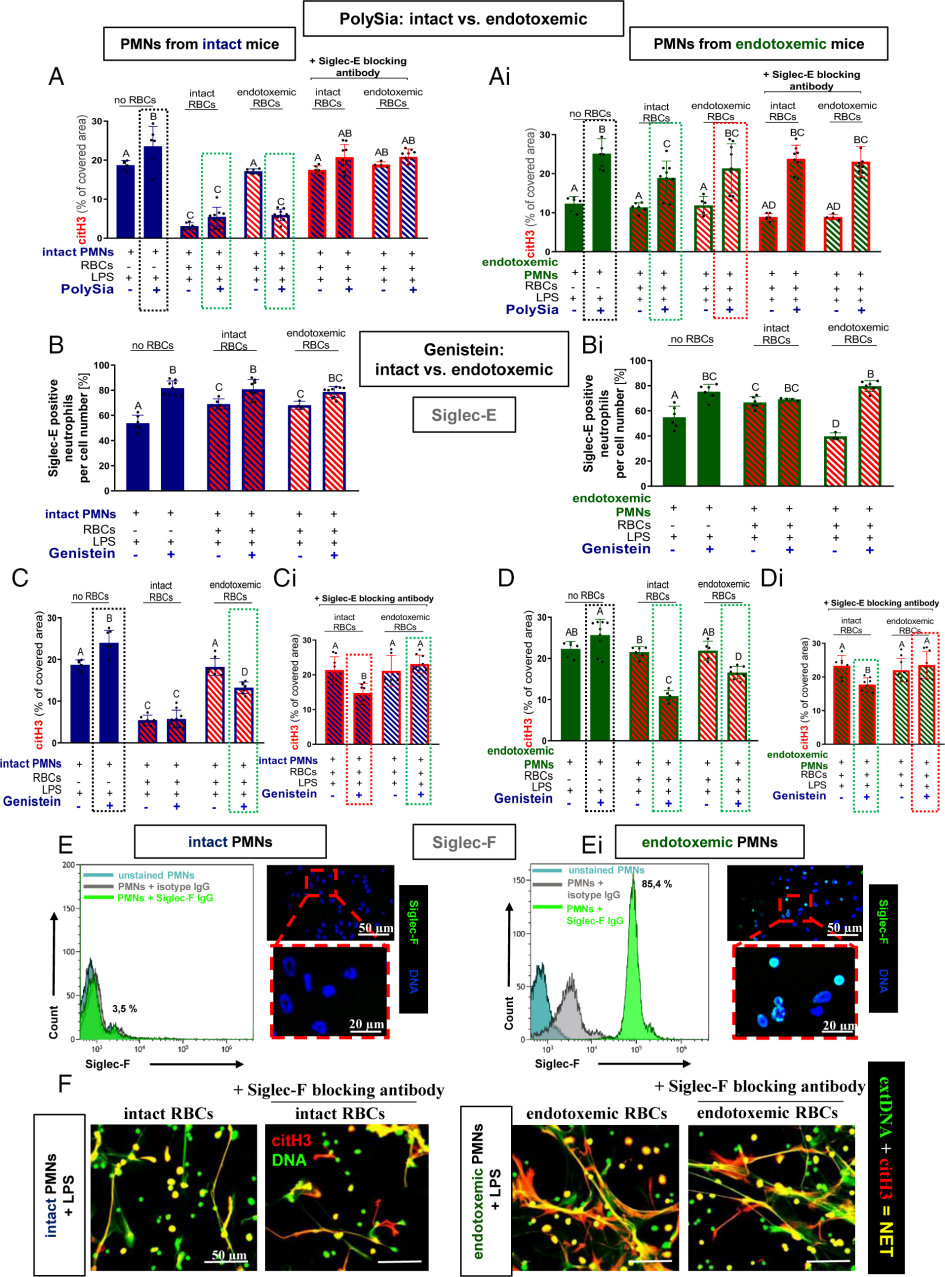
PolySia and restored Siglec-E expression, but not Siglec-F, partially reestablish inhibition of NET formation. Cells were isolated from healthy (intact) and endotoxemic mice and cultured as homo- or mixed RBC–PMN combinations. In selected experiments, polysialic acid (PolySia) and/or Siglec-E-blocking antibodies were added before LPS stimulation. NET formation (citH3) in intact vs. endotoxemic conditions with PolySia is shown in (*A* and *Ai*). Siglec-E expression on neutrophils was analyzed with or without genistein pretreatment (*B* and *Bi*). NET formation was assessed without or with Siglec-E blockade (*C*–*Di*). Siglec-F expression was evaluated by flow cytometry and confocal microscopy in intact and endotoxemic neutrophils (*E* and *Ei*), followed by analysis of NET formation under Siglec-F blockade (*F*). Results are presented as mean ± SD from at least three FOV. Statistically significant differences were marked with letter symbols according to one-way ANOVA (Bonferroni post hoc); n ≥ 3; PMNs-neutrophils, RBCs-erythrocytes.

We hypothesized that if the loss of RBCs’ ability to control neutrophil NET release results from a loss of sialic acid, exogenous sialic acid addition might restore this function. Indeed, the addition of PolySia to cocultures of endotoxemic (hence: desialylated) erythrocytes with intact neutrophils restored NET inhibition in a Siglec-E-dependent manner (Fig. 4*A*). However, in endotoxemic neutrophils, inhibition was not achieved; instead, NET release was increased (Fig. 4*Ai*). This indicates that the problem in RBC–PMN interactions also applies to neutrophils. A limitation of experiments with PolySia is that Siglec-E preferentially binds α2,3 rather than α2,8 residues, but we showed indirectly that it binds to erythrocytes, as the TER-119 signal was diminished (epitope masking) when PolySia was added to either intact or endotoxemic cells (*SI Appendix*, Fig. S11).

### Pharmacologically Augmented Expression of Siglecs on Endotoxemic Neutrophils Restores NET Suppression by Intact Erythrocytes.

If the restoration of sialic acid residues on RBCs cannot fully reestablish communication with endotoxemic neutrophils, we aimed to increase their Siglec-E expression. Genistein, a tyrosine kinase inhibitor, has previously been shown to block Siglec-8 clathrin-independent endocytosis, thereby preventing recycling to the plasma membrane and promoting its accumulation on the cell surface (17). Indeed, genistein increased Siglec-E expression on both intact and endotoxemic neutrophils (Fig. 4 *B* and *Bi*). Importantly, this effect was most pronounced when endotoxemic neutrophils were incubated with endotoxemic erythrocytes (Fig. 4*Bi*). In the coculture of intact cells, NET inhibition was maximal and unaffected by genistein (Fig. 4*C*). However, upon increased Siglec expression, even endotoxemic RBCs could somewhat inhibit NETs. Consistent with these data, despite preserved accumulation of Siglec-E molecules on endotoxemic neutrophils, addition of RBC from either intact or endotoxemic mice significantly downregulated NET release in a Siglec-E-dependent manner (Fig. 4*D*). However, the addition of genistein to endotoxemic neutrophils was not fully Siglec-E-dependent (Fig. 4 *Di*, *Right*). Moreover, although genistein did not further decrease NET release in a tandem of intact PMNs and intact RBCs, the Siglec-E blockade did (Fig. 4 *Ci*, *Left*). The latter data indicate that molecules other than Siglec-E may also be involved in NET inhibition, and that genistein unmasked this involvement.

### Siglec-G, but Not Siglec-F, Is Also Involved in RBC-Mediated NET Inhibition.

Siglec-F is also strongly associated with neutrophils (18). However, its expression was low on intact neutrophils (app. 3.5%) but increased during systemic inflammation (85%) (Fig. 4*E* vs. Fig. 4*Ei*). But blockage of Siglec-F revealed no impact on NET formation in the presence of RBCs (Fig. 4*F*).

Then we verified Siglec-G; however, its expression was primarily reported on T and B cells, with contradictory data on neutrophils (19). We demonstrated that only app. 20% of PMNs carry surface Siglec-G, and its expression was decreased on endotoxemic neutrophils (*SI Appendix*, Fig. S12 *A*, *Left* vs. *Right*). However, genistein dramatically increased Siglec-G accumulation on intact and endotoxemic PMNs (by up to 80% and 60%, respectively) (*SI Appendix*, Fig. S12 *A* and *Ai*). Sialic acid is a ligand of Siglec-G, but in myeloid cells, it can also form a ternary complex with antigens and CD24. However, CD24 blockade did not affect RBC-mediated NET regulation (*SI Appendix*, Fig. S12 *B* and *Bi*), and only when we also directly blocked Siglec-G was NET inhibition by RBCs interacting with endotoxemic neutrophils upregulated again (*SI Appendix*, Fig. S12*Ci*). Interestingly, blocking Siglec-G on intact neutrophils increased NET release, but only when genistein was applied (*SI Appendix*, Fig. S12*C*).

### Pharmacologically Augmented Expression of Siglec-E and -G on Endotoxemic Neutrophils and Addition of PolySia Restores NET Suppression by Erythrocytes.

**Fig. 5.**
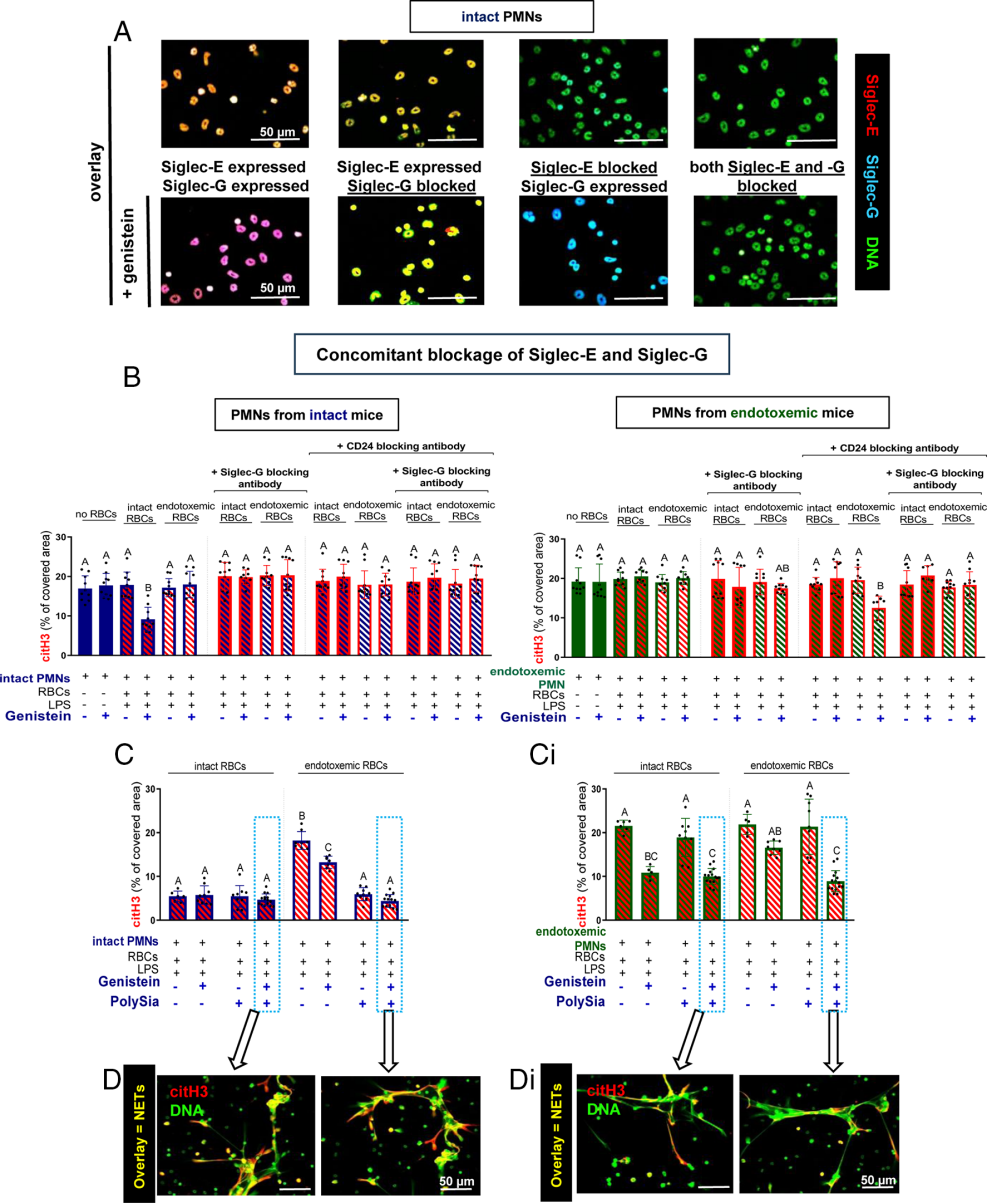
Concomitant application of genistein and PolySia sustains the inhibitory effect of erythrocytes, preventing extensive NET formation under endotoxemic conditions. Cells were isolated from healthy (intact) and endotoxemic mice, with some animals pretreated with Siglec-E-blocking antibodies and/or genistein. Ex vivo, cells were stimulated with LPS, with additional blockade of Siglec-G or CD24 in selected experiments. Siglec-E and -G expression was analyzed (*A*). NET formation (citH3) was assessed under different blocking conditions (*B*) and in cocultures or mixed cell populations from intact and endotoxemic mice, with or without PolySia (*C* and *Ci*). Representative images are shown in (*D* and *Di*). In some graphs, the blue frames indicate successful NET inhibition in the presence of PolySia and genistein. Results are presented as mean ± SD from at least three FOV. Statistically significant differences were marked with letter symbols according to one-way ANOVA (Bonferroni post hoc); n ≥ 3; PMNs-neutrophils, RBCs-erythrocytes.

To verify whether RBC-mediated regulation of NET release depends on Siglec-E and Siglec-G, both receptors, as well as CD24, were blocked in intact and endotoxemic neutrophils (Fig. 5*B*). This concomitant treatment completely reversed the inhibitory capacity of RBCs toward NETs in endotoxemic neutrophils. There were only two exceptions when the inhibition was not achieved. The first is for intact PMNs/RBCs treated with genistein (Fig. 5 *B*, *Left*). This confirms that, under homeostatic conditions, Siglec-E/-G expression is appropriate and efficient for arresting NETs and should not be increased. The other exception was in the case of endotoxemic PMNs/RBCs treated with genistein (Fig. 5 *B*, *Right*). We hypothesized that, when Siglec-G and -E levels are again high on endotoxemic neutrophils (upon genistein), the issue of low sialic acid expression on RBCs remains limiting. To address this, the RBC–PMN cocultures were treated with both genistein and PolySia (Fig. 5 *C*–*Di*). Consistently, this treatment led to maximal NET inhibition, regardless of cell origin. Importantly, only with combined treatment did endotoxemic erythrocytes inhibit NET release by endotoxemic neutrophils (Fig. 5*Ci*, yellow mask).

## Discussion

Despite emerging data, the relevance of erythrocyte–neutrophil interactions under physiological and pathophysiological conditions remains insufficiently understood. From an evolutionary perspective, the presence of immunomodulatory functions in mammalian erythrocytes aligns with the immune roles preserved in the nucleated counterparts of nonmammalian vertebrates, in which erythrocytes upregulate immune-related genes involved in antiviral defense and inflammatory responses, produce cytokine-like molecules, and engage in pathogen recognition/clearance (20).

Consistent with this, intact RBCs regulate neutrophil function in homeostasis, as previously shown for human cells and confirmed here for murine polymorphonuclear granulocytes. In particular, we confirmed that RBCs collected from healthy individuals regulate the viability and activation of neutrophils, as well as their effector functions, including PAD4-dependent NET formation in response to an acute insult (a single LPS bolus). Notably, increasing erythrocyte abundance (1:1,000 ratio) resulted in a dose-dependent enhancement of neutrophil viability and suppression of NET formation, with near-complete inhibition observed at higher ratios, which limited further mechanistic resolution. It should also be stressed that the present system does not capture additional layers of regulation present in circulation, including plasma sialoglycoproteins, endothelial glycocalyx, and flow-dependent forces. This will require in vivo studies to elucidate. However, it also allows the isolation of erythrocyte-derived sialylation as a discrete regulatory input. Our experiments were performed under consistent anticoagulation conditions, allowing direct comparison between experimental groups, although anticoagulant-specific effects cannot be formally excluded.

The important finding was that the erythrocyte-neutrophil cross-talk does not depend on LPS recognition (CD14-TLR4). Nor does this depend on RBC sequestration of LPS, despite its reported occurrence in other species (15). Our study confirms, however, that LPS does affect RBC condition, leading to the removal of surface sialic acids, as observed in endotoxemic erythrocytes. It was proposed that, within the organism, LPS acts via TLR4 signaling to increase the surface expression and activity of host sialidases that remove sialic acid residues from glycoproteins (21). In particular, previous studies have shown that lysosomal neuraminidase Neu1 can be rapidly mobilized to the plasma membrane upon LPS stimulation, inducing remodeling of cell-surface glycoconjugates (22). The same applies to Neu3 (23). Such mechanisms may contribute to the pronounced loss of erythrocyte sialylation (22, 24). Consistent with this, our mass spectrometry analyses confirmed that desialylation achieved during endotoxemia in vivo was very similar to that achieved ex vivo with *V. cholerae* sialidase treatment. Changes in RBC rheology, such as alterations in intracellular calcium and ATP concentrations, and a decrease in some RBC membrane components, such as sialic acid, and an increase in others, such as 2,3-diphosphoglycerate, are characteristic of sepsis (25). Notably, both α2,3- and α2,6-linked sialic acids have been reported to increase on the surface of innate immune cells upon LPS stimulation, indicating that also α2,6-linked sialoglycans are dynamically regulated by endotoxin (26). However, in our studies, on both human and murine erythrocytes, we mostly detected changes in α2,3-linked sialic acids.

Furthermore, we report that the neutrophil–erythrocyte interaction depends on the presence of sialic acids attached to the extracellular domains of RBC glycoproteins, as shown by *V. cholerae* sialidase- and Neuraminidase A-mediated removal of SiA when erythrocytes were collected from healthy individuals, and by the lack of such interactions during systemic inflammation. As previously shown for human neutrophils, Siglec-9 is essential for these interactions (5); we confirmed this in our system and further showed that the same operates in mice and relies on Siglec-E, the functional equivalent of the murine receptor (27). Importantly, we show that in neutrophils collected from healthy individuals, but not those with endotoxemia, SHP-1 is recruited to Siglec-E, confirming the downstream signaling. However, when at least one cell type was collected from either endotoxemic mice or septic patients, no NET inhibition was observed; it was restored during the recovery phase several days after the onset of systemic inflammation, in line with reports showing dysregulated Siglec-9-dependent neutrophil responses during sepsis and their normalization during recovery (28). This is consistent with the robust formation of NETs in blood during various human disorders, such as sepsis (29), COVID-19 (30), ischemic stroke, and cancer-associated thrombosis (31), as well as in murine sepsis models (32). If erythrocytes could control NET formation during blood-borne inflammation, robust NET release would not occur.

Of note, packed RBC transfusions have been shown to modulate neutrophil activation beyond their effects on oxygen delivery. In sickle cell disease, transfusions reduce neutrophil degranulation and oxidative burst, suggesting that erythrocytes can directly regulate neutrophil effector functions. In this context, restoration of erythrocyte sialylation and Siglec-dependent inhibitory signaling may additionally contribute to these effects (33).

Considering our findings, we aimed to restore and preserve sialic acids on RBCs and Siglecs on neutrophils during systemic inflammation. Polysialic acids (PolySia) are negatively charged carbohydrates consisting of α2,8-linked N-acetylneuraminic acid residues in mammals (34). Nevertheless, PolySia-coated nanoparticles enhanced oligomerization of murine Siglec-E receptors (which preferentially bind α2,3 residues) on the surface of macrophages, leading to an anti-inflammatory phenotype (35). PolySia is evolutionarily conserved and is among the most biologically essential glycoproteins in vertebrates (34). Additionally, it was reported that sialic acid-decorated nanoparticles abrogate sepsis via Siglec-dependent interactions (35). In the context of NETs, it is essential that PolySia chains can neutralize histone-mediated cytotoxicity (36). NETs contain histones that are released over time (3), which are among the major causes of death in sepsis (37). Critically, we showed that in vivo NET histones are covered by NE and are only gradually exposed over 2 wk, at which time they cleave Factor VII Activating Protease (FSAP), thereby activating it (3). Thus, FSAP activity in the presence of PolySia should be tested in this model. It might be that PolySia will also inhibit NET cytotoxicity in the above time frame. If so, PolySia may act at the level of NET generation and by modulating the activity of already released NET components (38, 39). Interestingly, PolySia is present in human plasma under homeostatic conditions (39), but it is unknown whether this changes during sepsis. Importantly, nanoparticles equipped with α2,8-linked sialic acid chains inhibited NET release (40).

Indeed, the addition of exogenous PolySia to cocultures with endotoxemic RBCs and intact neutrophils restored NET inhibition. Given its beneficial effects on erythrocyte responsiveness under endotoxemic conditions, PolySia’s biocompatibility and biodegradability (41) are important for possible therapeutic applications. Moreover, the latter might be replaced with PolySia-coated nanoparticles or other polymers enriched in α2,3 residues. Overall, our findings align with studies showing that sialic acid-decorated nanoparticles and synthetic glycopolymers suppress inflammation via Siglec engagement, dependent on ligand density and multivalent presentation (35, 42). In this context, the partial activity of PolySia observed in our system supports the idea that effective NET inhibition requires surface-associated sialylated ligands, rather than the soluble form.

On the other hand, when high Siglec-E expression on neutrophils was preserved with genistein, endotoxemic neutrophils responded to intact RBCs, producing fewer NETs. Genistein is a plant-derived tyrosine kinase inhibitor that prevents Siglec receptor recycling and promotes their accumulation on the cell surface; however, given its broad activity, the effects cannot be attributed exclusively to Siglec-E-dependent signaling (43). Genistein is not selective for individual Siglecs, and no selective compounds exist. Although genistein may affect other neutrophil receptors, including L-selectins, Fc receptors, and integrins (44), these are not known to mediate neutrophil–erythrocyte interactions. Importantly, comparable effects on NET formation were observed with selective anti-Siglec-E and anti-Siglec-G antibodies, supporting their involvement, although a broader receptor modulation cannot be excluded and should be addressed in vivo. Importantly, genistein has been shown to have overall beneficial effects during systemic inflammation: it improved outcomes in sepsis-associated encephalopathy (45) and mitigated vascular dysfunction during endotoxemia (46). In clinical trials, orally administered genistein entered phase II testing for bladder cancer (47) but was not successful in septic patients when evaluated for cytokine inhibition (36), although it was successful in vitro (48).

Given the beneficial effects of PolySia on endotoxemic erythrocytes and of genistein on endotoxemic neutrophils, both agents were combined in neutrophil–erythrocyte cultures. This resulted in the complete restoration of NET inhibition. To fully confirm these observations, we additionally blocked Siglec-E to verify that interaction with sialic acids was not compromised. Whereas it was indeed the case for endotoxemic RBCs and endotoxemic neutrophils, in other cellular combinations (intact cells or endotoxemic PMNs with intact RBCs), NET inhibition remained partially preserved despite Siglec-E blockade, suggesting involvement of additional Siglec receptors. Hence, we verified the expression of additional Siglec molecules reported in the literature to have anti-inflammatory characteristics. Siglecs-F/8/5 were reported to be expressed on neutrophils (18); however, blockage of these receptors in the context of RBC–PMN interactions did not inhibit NETs. Subsequently, we focused on Siglec-G, expressed on macrophages, and weakly on T cells (19), although data on neutrophils were incoherent (49). We confirmed its expression on PMNs, but it was low; however, genistein dramatically increased it. This suggests that neutrophils express Siglec-G transcripts and can upregulate the receptor in response to extracellular cues. Under these conditions, enhanced surface availability of Siglec-G contributes to NET inhibition, consistent with increased responsiveness to sialylated ligands, whereas its role under baseline conditions appears limited compared to Siglec-E.

In summary, we report that endogenous NET-controlling mechanisms based on the erythrocyte Siglec-sialic acid axis, which operate in homeostasis but are disrupted during systemic inflammation, can be pharmacologically restored. As such, the data propose a possible therapeutic approach to control excessive NET release in the vasculature, which otherwise contributes to the inflammation-related pathogenesis of sepsis.

## Materials and Methods

Additional description of methods is presented in *SI Appendix*, along with *SI Appendix*, Figs. S1–S12 and Table S1 and Dataset S1.

### Mice.

C57BL/6J male mice (6 to 12 wk old) were purchased from Charles River Laboratories (Sulzfeld, Germany, via AnimaLab). Peptidyl arginine deiminase 4 knockout mice (PAD4 KO) were purchased from the Jackson Laboratory (Bar Harbor, ME). The animals were maintained at 21 to 22 °C under a 12-h light/dark cycle. The food and tap water were available ad libitum. All experimental animal protocols were approved by the Local Ethical Committee No. II in Krakow, Poland (permission Nos. 22/2023, 22A/2023), ensuring compliance with EU Animal Care Guidelines.

### Human Samples.

The human study was approved by the Bioethics Committee of Jagiellonian University in Krakow (permission No. 1072.6120.258.2019). Adult blood donors (healthy volunteers and patients with sepsis or Lyme disease) aged 28 to 88 y were eligible to participate in this study. Blood samples from septic patients were collected at admission (1 to 2 d) and after 10 to 14 d of treatment (recovery phase).

### Induction of Endotoxemia/Systemic Inflammation in Mice.

Mice were intraperitoneally (i.p.) injected with 1 mg/kg per body weight (b.w.) lipopolysaccharide (LPS, **P. aeruginosa*,* serotype 10.22; Sigma‐Aldrich, Saint Louis, MO) in 0.9% saline (NaCl) to induce endotoxemia. Samples were collected 8 h or 6 d after LPS administration, as detailed below. Some of their counterparts remained untreated (intact controls).

### Murine or Human RBC–Neutrophil Cocultures.

The experiments were performed in 96-well plates. Where indicated, 5-mm coverglasses were placed at the bottom of the wells before neutrophil seeding (50,000 cells/well), followed by a 30-min incubation (37 °C, 5% CO_2_) to allow cell adhesion. In selected experiments (as indicated in the figures), intact or endotoxemic neutrophils were cocultured with RBCs from both intact and endotoxemic mice. The same setup was applied to human cells. In additional studies, RBCs and neutrophils were isolated from mice at the recovery phase of endotoxemia (6 d post-LPS) and incorporated into cocultures with intact, endotoxemic, or recovery-state cells. Analogously, human cells from the recovery phase of sepsis (10 to 14 d after diagnosis) were cocultured with autologous cells. Detailed protocols for cell isolation are described in *SI Appendix*.

### Ex Vivo LPS Treatment of Human or Murine Cocultures.

RBC–neutrophil cocultures were stimulated for 6 h (murine cells) or 3 h (human cells) with LPS (**P. aeruginosa*,* serotype 10.22; Sigma‐Aldrich, Saint Louis, MO) at a final well concentration of 50 μg/mL.

### Verification of LPS Uptake by Erythrocytes in Neutrophil Murine Cocultures.

To determine whether the erythrocyte-dependent inhibition of NET release resulted from sequestration of LPS by RBC, LPS uptake was assessed in monocultures and PMN-RBC cocultures using Alexa Fluor 568-conjugated LPS (*E*. *coli* O55:B5; Invitrogen/ThermoFisher Scientific, Waltham, MA; 10 µg/mL, for 6 h), as detailed in *SI Appendix*. Localization and cellular uptake of LPS were visualized by confocal microscopy, and LPS-positive cells were quantified as the percentage of LPS-binding and/or internalizing cells. Where indicated, cells were pretreated with neutralizing antibodies against TLR4/MD-2 or CD14, with isotype-matched controls. Detailed protocols for LPS uptake and measurements of other neutrophil effector functions are described in *SI Appendix*.

### Murine or Human Inhibitors of Siglec Receptors.

To determine whether RBC–PMN interactions are mediated by Siglec receptors, inhibitors of Siglec-E/-G/-F (murine) or Siglec-9 (human) receptors were applied 30 min before RBCs and/or LPS addition. Isotype-matched antibodies were used as controls, as described in *SI Appendix*. To assess potential compensation between Siglec-E and Siglec-G, mice received an intravenous (i.v.) injection of anti-Siglec-E neutralizing antibody (25 mg/kg b.w. in 0,9% NaCl) 1 h before endotoxemia induction. Inhibitor efficacy was assessed by immunocytochemistry for Siglec-E/-G or Siglec-9 expression. Detailed protocols are described in *SI Appendix*.

### Murine or Human RBCs Treatment with Sialidases.

To investigate whether Siglec receptors interfere with sialic acid residues, RBCs were treated with sialidases (0.125 U; neuraminidase from *V. cholerae*; Roche, Basel, Switzerland, or 100 U; α2-3,6,8,9 Neuraminidase A; New England BioLabs, United States) for 1 h before coculture with neutrophils to remove surface sialic acid residues. Cells were centrifuged (1,500 rcf, 22 °C, 5 min), resuspended in HBSS (+), and washed twice to remove residual enzyme and sialic acids. The efficiency of desialylation was verified by lectin-based flow cytometry using biotinylated MAL II and SNA (5 µg/mL in 1% BSA in PBS; Vector Laboratories, United States). RBCs were incubated with lectins (1 h, 4 °C), followed by DyLight 649-conjugated streptavidin incubation (1 µg/mL in 1% BSA in PBS; Vector Laboratories, United States) for 1 h at 4 °C. Samples were analyzed using a Navios flow cytometer (Beckman Coulter, Brea, CA).

### Exogenous Sialic Acids (PolySia) Treatment of Murine Cocultures.

To evaluate the effects of exogenous sialic acid residues on neutrophil responses, neutrophils from intact or endotoxemic mice (8 h) were seeded onto 96-well plates containing 5-mm coverslips and preincubated for 30 min with colominic acid sodium salt (10 µg/mL; PolySia; MedChemExpress, New Jersey, NJ), at 37 °C and 5% CO_2_. Next, either intact or endotoxemic erythrocytes and/or LPS were added, and cultures were maintained for 6 h. Parallel control groups without PolySia enabled assessment of PolySia-dependent effects. Cells were fixed and processed for immunocytochemical analysis of NET formation. In additional experiments, RBCs were incubated with PolySia (10 µg/mL) for 6 h (37 °C, 5% CO_2_) to assess its association with the erythrocyte surface. Following incubation, RBCs were stained with AlexaFluor 647 anti-mouse TER-119 antibody (clone TER-119, BioLegend, San Diego, CA), cytospins were prepared, and samples were analyzed by confocal microscopy. Detailed protocols for immunocytochemical staining and microscopic analyses are described in *SI Appendix*.

### Genistein Treatment to Enhance Siglecs Expression on Murine Leukocytes.

To increase the surface expression of Siglec-E and Siglec-G on murine leukocytes, genistein was administered in selected experiments 8 h before endotoxemia induction. Mice received an intraperitoneal injection of genistein (150 mg/kg b.w.) in a vehicle containing 10% DMSO (Sigma-Aldrich, Saint Louis, MO), 45% PEG300 (MedChemExpress, New Jersey, NJ), and 45% NaCl. Control animals received the vehicle alone. Isolated cells were used for downstream analyses and cocultures as described in “*Murine or Human RBC–Neutrophil Cocultures.*”

### Characterization of Erythrocyte Surface *N*-Glycans by MALDI-ToF-MS.

To assess changes in erythrocyte surface sialylation during homeostasis and endotoxemia (8 h and 6 d), *N*-glycan profiling was performed using MALDI-TOF-MS, as described in *SI Appendix*. Erythrocytes were isolated from cardiac blood, adjusted to 5 × 10^8^ cells/sample, and resuspended in 200 μL HBSS (+). Where indicated, cells were treated with 0.125 U sialidase (*V. cholerae*) for 1 h (37 °C, 5% CO_2_) or vehicle control, followed by washing. Analyses were performed on a rapifleX™ MALDI-TOF mass spectrometer (Bruker Daltonics, Bremen, Germany) operating in positive-ion reflectron mode (m/z 500 to 5,000; 32,000 shots/sample), with external calibration using Peptide Calibration Mix II. N-glycan peaks were assigned based on m/z values, and glycan structures were visualized using GlycoWorkbench platform. Detailed protocols are described in *SI Appendix*.

### Statistical Analyses.

All data are presented as mean values ± SD. The Shapiro–Wilk test was performed to determine the normality of the data. Data were compared either by using the parametric unpaired two-tailed Student’s *t* test or one-way ANOVA with the Bonferroni multiple comparisons post hoc test. Statistical significance was set at *P* < 0.05.

## Supplementary Material

Appendix 01 (PDF)

Dataset S01 (PDF)

## Data Availability

Study data are included in the article and/or supporting information.

## References

[1] E. Kolaczkowska , Molecular mechanisms of NET formation and degradation revealed by intravital imaging in the liver vasculature. Nat. Commun. **6**, 1–13 (2015). 10.1038/ncomms7673 PMC4389265 25809117

[2] B. McDonald, R. Davis, S.-J. Kim, M. Tse, Platelets and neutrophil extracellular traps collaborate to promote intravascular coagulation during sepsis in mice. Blood **129**, 1357–1367 (2017). 28073784 10.1182/blood-2016-09-741298 PMC5345735

[3] M. Santocki, A. Such, D. Drab, G. Burczyk, E. Kolaczkowska, NETs persisting in vasculature undergo self-renewal with consequences for subsequent infection: A mouse model study. Blood **145**, 2070–2085 (2025). 39841013 10.1182/blood.2024026643 PMC12105727

[4] C. C. Yost , Neonatal NET-inhibitory factor and related peptides inhibit neutrophil extracellular trap formation. J. Clin. Invest. **126**, 3783–3798 (2016). 27599294 10.1172/JCI83873 PMC5096809

[5] A. Lizcano , Erythrocyte sialoglycoproteins engage Siglec-9 on neutrophils to suppress activation. Blood **129**, 3100–3110 (2017). 28416510 10.1182/blood-2016-11-751636 PMC5465837

[6] E. Karsten, E. Breen, B. R. Herbert, Red blood cells are dynamic reservoirs of cytokines. Sci. Rep. **8**, 1–12 (2018). 29449599 10.1038/s41598-018-21387-w PMC5814557

[7] M. J. Hotz , Red blood cells homeostatically bind mitochondrial DNA through TLR9 to maintain quiescence and to prevent lung injury. Am. J. Respir. Crit. Care Med. **197**, 470–480 (2018). 29053005 10.1164/rccm.201706-1161OC PMC5821907

[8] J. Baum, R. H. Ward, D. J. Conway, Natural selection on the erythrocyte surface. Mol. Biol. Evol. **19**, 223–229 (2002). 11861881 10.1093/oxfordjournals.molbev.a004075

[9] V. Kuhn , Red blood cell function and dysfunction: Redox regulation, Nitric Oxide metabolism, Anemia. Antioxid. Redox Signal. **26**, 718–742 (2017). 27889956 10.1089/ars.2016.6954 PMC5421513

[10] R. M. Bateman, M. D. Sharpe, M. Singer, C. G. Ellis, The effect of sepsis on the erythrocyte. Int. J. Mol. Sci. **18**, 1932 (2017). 28885563 10.3390/ijms18091932 PMC5618581

[11] A. F. Carlin , Molecular mimicry of host sialylated glycans allows a bacterial pathogen to engage neutrophil Siglec-9 and dampen the innate immune response. Blood **113**, 3333–3336 (2009). 19196661 10.1182/blood-2008-11-187302 PMC2665898

[12] A. Varki, Uniquely human evolution of sialic acid genetics and biology. Proc. Natl. Acad. Sci. U.S.A. **107**, 8939–8946 (2010). 20445087 10.1073/pnas.0914634107 PMC3024026

[13] W. Ortmann , Large extracellular vesicle (EV) and neutrophil extracellular trap (NET) interaction captured in vivo during systemic inflammation. Sci. Rep. **14**, 1–20 (2024). 38409254 10.1038/s41598-024-55081-x PMC10897202

[14] D. J. Gregory , Molecular profiles of blood from numerous species that differ in sensitivity to acute inflammation. Mol. Med. **30**, 280 (2024). 39730996 10.1186/s10020-024-01052-x PMC11681734

[15] C. Carr, D. C. Morrison, Lipopolysaccharide interaction with rabbit erythrocyte membranes. Infect. Immun. **43**, 600–606 (1984). 6693170 10.1128/iai.43.2.600-606.1984 PMC264341

[16] S. O. Vasudevan, A. J. Russo, P. Kumari, S. K. Vanaja, V. A. Rathinam, A TLR4-independent critical role for CD14 in intracellular LPS sensing. Cell Rep. **39**, 110755 (2022). 35508125 10.1016/j.celrep.2022.110755 PMC9376664

[17] J. A. O’Sullivan, D. J. Carroll, Y. Cao, A. N. Salicru, B. S. Bochner, Leveraging Siglec-8 endocytic mechanisms to kill human eosinophils and malignant mast cells. J. Allergy Clin. Immunol. **141**, 1774–1785 (2018). 28734845 10.1016/j.jaci.2017.06.028 PMC6445644

[18] S. Ryu , Siglec-F-expressing neutrophils are essential for creating a profibrotic microenvironment in renal fibrosis. J. Clin. Invest. **132**, 17–19 (2022). 10.1172/JCI156876 PMC9197522 35482420

[19] W. Royster, P. Wang, M. Aziz, The role of Siglec-G on immune cells in sepsis. Front. Immunol. **12**, 1–12 (2021). 10.3389/fimmu.2021.621627 PMC7940683 33708213

[20] H. L. Anderson, I. E. Brodsky, N. S. Mangalmurti, The evolving erythrocyte: Red blood cells as modulators of innate immunity. J. Immunol. **201**, 1343–1351 (2018). 30127064 10.4049/jimmunol.1800565 PMC6108441

[21] D. H. Allendorf, E. H. Franssen, G. C. Brown, Lipopolysaccharide activates microglia via neuraminidase 1 desialylation of Toll-like Receptor 4. J. Neurochem. **155**, 403–416 (2020). 32279315 10.1111/jnc.15024

[22] M. A. Aljohani, H. Sasaki, X. L. Sun, Cellular translocation and secretion of sialidases. J. Biol. Chem. **300**, 107671 (2024). 39128726 10.1016/j.jbc.2024.107671 PMC11416241

[23] F. D’Avila , Identification of lysosomal sialidase NEU1 and plasma membrane sialidase NEU3 in human erythrocytes. J. Cell. Biochem. **114**, 204–211 (2013). 22903576 10.1002/jcb.24355

[24] S. R. Amith , Neu1 desialylation of sialyl α-2, 3-linked β-galactosyl residues of TOLL-like receptor 4 is essential for receptor activation and cellular signaling. Cell. Signal. **22**, 314–324 (2010). 19796680 10.1016/j.cellsig.2009.09.038

[25] M. Piagnerelli, K. Zouaoui Boudjeltia, M. Vanhaeverbeek, J. L. Vincent, Red blood cell rheology in sepsis. Intensive Care Med. **29**, 1052–1061 (2003). 12802488 10.1007/s00134-003-1783-2

[26] Y. Wu, C. Lan, D. Ren, G. Y. Chen, Induction of siglec-1 by endotoxin tolerance suppresses the innate immune response by promoting TGF-β1 production. J. Biol. Chem. **291**, 12370–12382 (2016). 27129263 10.1074/jbc.M116.721258 PMC4933283

[27] S. J. McMillan , Siglec-E is a negative regulator of acute pulmonary neutrophil inflammation and suppresses CD11b b2-integrin-dependent signaling. Blood **121**, 2084–2094 (2013). 23315163 10.1182/blood-2012-08-449983 PMC3596968

[28] S. Von Gunten, S. M. Jakob, B. Geering, J. Takala, H. U. Simon, Different patterns of SIGLEC-9-mediated neutrophil death responses in septic shock. Shock **32**, 386–392 (2009). 19295491 10.1097/SHK.0b013e3181a1bc98

[29] H. Zhang , Ferritin-mediated neutrophil extracellular traps formation and cytokine storm via macrophage scavenger receptor in sepsis-associated lung injury. Cell Commun. Signal. **22**, 1–17 (2024). 38308264 10.1186/s12964-023-01440-6 PMC10837893

[30] Y. Zuo , Neutrophil extracellular traps in COVID-19. JCI Insight **9**, 1–18 (2020). 10.1172/jci.insight.138999 PMC7308057 32329756

[31] X. Xu , Clinical significance of neutrophil extracellular traps biomarkers in thrombosis. Thromb. J. **20**, 1–17 (2022). 36224604 10.1186/s12959-022-00421-y PMC9555260

[32] K. Tanaka , In vivo characterization of neutrophil extracellular traps in various organs of a murine sepsis model. PLoS One **9**, e111888 (2014). 25372699 10.1371/journal.pone.0111888 PMC4221155

[33] K. E. Remy , Mechanisms of red blood cell transfusion-related immunomodulation. Transfusion **58**, 804–815 (2018). 29383722 10.1111/trf.14488 PMC6592041

[34] C. Sato, K. Kitajima, Disialic, oligosialic and polysialic acids: Distribution, functions and related disease. J. Biochem. **154**, 115–136 (2013). 23788662 10.1093/jb/mvt057

[35] S. Spence , Targeting Siglecs with a sialic acid-decorated nanoparticle abrogates inflammation. Sci. Transl. Med. **7**, 1–13 (2015). 10.1126/scitranslmed.aab3459 26333936

[36] C. E. Galuska , Artificial polysialic acid chains as sialidase-resistant molecular-anchors to accumulate particles on neutrophil extracellular traps. Front. Immunol. **8**, 2–11 (2017). 29033944 10.3389/fimmu.2017.01229 PMC5626807

[37] J. Xu , Extracellular histones are major mediators of death in sepsis. Nat. Med. **15**, 1318–1321 (2009). 19855397 10.1038/nm.2053 PMC2783754

[38] K. Zlatina, T. Lütteke, S. P. Galuska, Individual impact of distinct polysialic acid chain lengths on the cytotoxicity of histone H1, H2A, H2B, H3 and H4. Polymers (Basel) **9**, 1–13 (2017). 30966022 10.3390/polym9120720 PMC6418544

[39] K. Zlatina , Polysialic acid in human plasma can compensate the cytotoxicity of histones. Int. J. Mol. Sci. **19**, 1679 (2018). 29874880 10.3390/ijms19061679 PMC6032143

[40] K. F. Bornhöfft, T. Viergutz, A. Kühnle, S. P. Galuska, Nanoparticles equipped with α2, 8-linked sialic acid chains inhibit the release of neutrophil extracellular traps. Nanomaterials **9**, 13–15 (2019). 10.3390/nano9040610 PMC6523985 31013834

[41] S. Berski , Synthesis and biological evaluation of a polysialic acid-based hydrogel as enzymatically degradable scaffold material for tissue engineering. Biomacromolecules **9**, 2353–2359 (2008). 18690740 10.1021/bm800327s

[42] C. S. Delaveris, S. H. Chiu, N. M. Riley, C. R. Bertozzi, Modulation of immune cell reactivity with cis-binding Siglec agonists. Proc. Natl. Acad. Sci. U.S.A. **118**, 1–11 (2021). 10.1073/pnas.2012408118 PMC7826350 33431669

[43] Y. X. Goh, J. Jalil, K. W. Lam, K. Husain, C. M. Premakumar, Genistein: A review on its anti-inflammatory properties. Front. Pharmacol. **13**, 1–23 (2022). 10.3389/fphar.2022.820969 PMC8818956 35140617

[44] J. Sharifi-Rad , Genistein: An integrative overview of its mode of action, pharmacological properties, and health benefits. Oxid. Med. Cell. Longev. **2021**, 3268136 (2021). 34336089 10.1155/2021/3268136 PMC8315847

[45] I. Passos , Polysialic acid restrains inflammatory monocyte maturation. Front. Immunol. **16**, 1–13 (2025). 10.3389/fimmu.2025.1656087 PMC12580094 41190064

[46] A. Bermejo, A. Zarzuelo, J. Duarte, In vivo vascular effects of genistein on a rat model of septic shock induced by lipopolysaccharide. J. Cardiovasc. Pharmacol. **42**, 329–338 (2003). 12960677 10.1097/00005344-200309000-00003

[47] E. Messing , A phase 2 cancer chemoprevention biomarker trial of isoflavone G-2535 (genistein) in presurgical bladder cancer patients. Cancer Prev. Res. **5**, 621–630 (2012). 10.1158/1940-6207.CAPR-11-0455 PMC3324663 22293631

[48] A. Matsumori , Modulation of cytokine production and protection against lethal endotoxemia by the cardiac glycoside ouabain. Circulation **96**, 1501–1506 (1997). 9315538 10.1161/01.cir.96.5.1501

[49] F. Pfrengle, M. S. Macauley, N. Kawasaki, J. C. Paulson, Copresentation of antigen and ligands of Siglec-G induces B cell tolerance independent of CD22. J. Immunol. **191**, 1724–1731 (2013). 23836061 10.4049/jimmunol.1300921 PMC3735655

