# Supplementary material for: Loss of erythrocyte sialic acid in sepsis disrupts inhibitory Siglec interactions, driving neutrophil hyperactivation and NET outspread
Source: Proc Natl Acad Sci U S A. 2026 Jun 10;123(24):e2536989123. doi: 10.1073/pnas.2536989123 (PMC13273359; doi:10.1073/pnas.2536989123)
Supplement: Supplementary file 1 — Appendix 01 (PDF) [file pnas.2536989123.sapp.pdf]

PNAS

Supporting Information for

Loss of erythrocyte sialic acid in sepsis disrupts inhibitory Siglec interactions, driving neutrophil hyperactivation and NET outspread

Anna Such, Weronika Ortmann, Gabriela Burczyk, Jacek Czepiel, Monika Bociaga-Jasik, Paweł Link-Lenczowski, Elzbieta Kolaczkowska*

*Corresponding author: Elzbieta Kolaczkowska, Laboratory of Experimental Hematology, Institute of Zoology and Biomedical Research, Faculty of Biology, Jagiellonian University, Gronostajowa 9, 30-387 Krakow, Poland

Email: *ela.kolaczkowska@uj.edu.pl*

This PDF file includes:

Supporting text
Figures S1 to S12
Table S1
Dataset S1
SI References

Supplementary Methods

Human samples – extended information

Adult blood donors admitted to the Infectious Diseases Clinical Department at the University Hospital in Krakow with a diagnosis of sepsis, bacteremia, or with symptoms of bacterial infection were eligible to participate in this study. The inclusion criteria for patients with sepsis were defined according to the Third International Consensus Definition for Sepsis (Sepsis-3) and included patients aged 28-88 years. The control group consisted of healthy volunteers who were age- and gender-matched to the septic patients. Additional control samples were collected from patients with a local infection (Lyme disease). Patient details are provided in Supplementary Table 1. Written informed consent was obtained from all participants.

Red blood cell (RBC) isolation from murine blood – extended information

The experiments were performed on red blood cells isolated from the whole blood of endotoxemic mice (8 h or 6 days after LPS administration) or unstimulated (intact) controls. Before RBC isolation animals were anesthetized by i.p. injection with a mixture of ketamine hydrochloride (200 mg/kg body weight; Biowet Pulawy, Pulawy, Poland) and xylazine hydrochloride (10 mg/kg b.w; antiMEDICA, Südfeld, Germany). Blood was collected by cardiac puncture using heparinized syringe and diluted in HBSS (-) to preserve cell viability and prevent coagulation. RBCs were separated by Percoll (GE Healthcare, Arlington Heights, IL, USA) density gradient centrifugation. Briefly, 3 mL of Percoll solutions at 65% and 72% were loaded into a glass tube. Diluted blood was gently layered onto the Percoll gradient, then centrifuged for 35 minutes at 22°C at 1,800 rpm. The erythrocyte pellet was suspended in HBSS (-) and centrifuged (2,500 rpm, 22°C, 6 min). Finally, the pellet was resuspended in 1 mL of HBSS (+), and RBCs were counted in a Bürker hemocytometer. In some initial experiments, cells were resuspended in RPMI 1640 or HBSS (-) (both from Lonza Bioscience, Basel, Switzerland) to compare cell viability depending on the medium used (Fig. S1A). RBCs purity was estimated as 99,6% with a flow cytometry (anti-TER-119; please compare in section Flow cytometry).

RBC isolation from human blood

RBCs were isolated from human blood using the same Percoll gradient and centrifugation conditions as for murine cells. Each procedure was performed on a fixed volume of 250 μL of whole blood.

Neutrophil isolation from murine bone marrow

Bone marrow (BM) neutrophils (PMNs) were isolated from mice as previously described (1). Briefly, mice were anesthetized with a ketamine-xylazine mixture as described above, and cervical dislocation was then performed. Tibias and femurs were collected, cleaned, and flushed with ice-cold HBSS (-). The bone marrow suspension was mechanically dispersed, centrifuged (1,300 rpm, 6 min, 4°C), and subjected to brief hypotonic lysis (0,2/1,6% NaCl) to remove erythrocytes. Cells were then separated using a three-layer Percoll gradient (52%, 69%, 78%), and the neutrophil fraction was collected, washed, and resuspended in HBSS (+). The cells were stained with Trypan blue dye (Sigma-Aldrich, Saint Louis, MO, USA) to assess viability and cell counts. Purity was determined upon Türk solution staining (0.01% crystal violet in 3% acetic acid; Sigma-Aldrich, Saint Louis, MO, USA) in a Bürker hemocytometer. The purity and viability of isolated neutrophils were ~99% and ~98%, respectively, as estimated by their morphology (Türk/Trypan blue staining), and numbers of Ly6G$^+$ cells (~70%, flow cytometry(1).

Human blood neutrophil isolation

A total of 3 mL of whole peripheral blood, anticoagulated with heparin, was diluted 1:1 with phosphate-buffered saline (PBS), layered over 4,5 mL of Biocoll (1,077 g/mL, Biochrom, Germany), and centrifuged at 1,600 rpm for 30 minutes (RT). The plasma layer and buffy coat were removed, and two rounds of hypotonic lysis with dH_2O were performed to eliminate erythrocytes. The cells were centrifuged (1,400 rpm, 21°C, 6 min), resuspended in HBSS (+), and counted using a Bürker hemocytometer, yielding a neutrophil purity of >98%.

Murine or human RBC-neutrophil co-cultures – extended information
Prior to LPS stimulation, RBCs were added at a PMN:RBC ratio of 1:4 (200,000 RBCs/well). The RBC concentration was chosen based on previous studies on human neutrophils (2). In some murine experiments, neutrophils were incubated with different RBC ratios (namely 1:40, 1:400, and 1:1000; Figure S2A-B). To determine whether the order of adding murine RBCs and neutrophils (PMNs) to the co-cultures influences neutrophil effector functions, two distinct cell-seeding strategies were evaluated (Fig. S1C-D). In the first approach, neutrophils were allowed to adhere for 30 minutes before the addition of RBCs, followed by simultaneous LPS stimulation (PMNs → RBCs + LPS). In the second approach, RBCs were seeded and incubated for 30 minutes at 37°C with 5% CO_2; after neutrophils were added, the cells were immediately stimulated with LPS (RBCs → PMNs + LPS). For experiments involving PAD4-deficient mice, RBCs and neutrophils were isolated exclusively from intact PAD4 KO and WT animals. Co-cultures were established by mixing KO neutrophils with WT RBCs or WT neutrophils with KO RBCs to assess the contribution of PAD4-dependent mechanisms to RBC-neutrophil interactions (Fig. S8). In selected human studies, an additional control group consisted of patients diagnosed with Lyme disease (a model of local inflammation), and, in selected experiments, co-cultures of RBCs and PMNs obtained from these individuals were also included (Fig. S9).

***Ex vivo* LPS treatment of human or murine co-cultures – extended information**
In some studies, murine cells were stimulated with LPS derived from *Escherichia coli* (serotype 0111:B4; Sigma–Aldrich, Saint Louis, MO, USA) at a concentration of 75 μg/mL to evaluate the impact of LPS source on neutrophil effector functions (Fig. S2C).

3D culturing of murine cells
For 3D culturing, PuraMatrix (#354250, Corning) at a final concentration of 0.25% (w/v) was diluted in 13.3% sucrose (1:3 proportion) and mixed according to the provider's specifications. To determine the physiological pH of the scaffolds, wells containing PuraMatrix were equilibrated with 120 μL of HBSS (+) overnight and then removed just before cell addition. The cells were seeded on top of the scaffolds at the same RBC-PMN ratio (4:1) as for 2D co-culture and were stimulated with LPS as previously described (Fig. S3). No encapsulation (entrapping of the cells inside of PuraMatrix) was performed, but with time, some cells migrated into the scaffolds. The latter phenomenon was thus mimicking *in vivo* neutrophil behavior.

***Ex vivo* LPS treatment of murine co-cultures – extended information**
To assess whether murine RBCs inhibit NET formation only when simultaneously co-incubated with LPS, we investigated the effect of RBC addition to neutrophils in a different order. Namely, RBCs were added to neutrophils 1 hour before LPS stimulation (PMNs → RBCs → LPS), or LPS was added 1 hour before RBCs (PMNs → LPS → RBCs) (Fig. S4).

Presto Blue viability assay
Cell viability was evaluated using the PrestoBlue® assay (Invitrogen, Waltham, MA, USA; Fig. S1-3). After 5.5 hours of incubation, 10 μl of PrestoBlue reagent (1:10 ratio) was added to each well, and the plate was incubated at 37°C with 5% CO_2 for an additional 30 minutes. Fluorescence was then measured at 535/595 nm using a spectrofluorometer (Infinite 200 Pro) 6 h after LPS addition. Results are expressed as fluorescence arbitrary units (A.U.).

Mitochondrial activity assay – MTT
Neutrophil metabolic activity was assessed using the MTT assay (Sigma–Aldrich, Saint Louis, MO, USA; Fig. S1 and Fig.S3). After a 3-hour incubation, 20 μl of MTT solution (5 mg/mL) was added to each well, followed by a further 3-hour incubation. Subsequently, the supernatant was carefully removed, and the formed formazan crystals were dissolved in 100 μl of 0.04 M HCl (Avantor, Poland) prepared in isopropanol (Chempur, Poland). The absorbance was then measured at 570 nm using an Infinite 200 Pro spectrophotometer.

Nitric oxide (NO) production assay

The neutrophil capacity to generate nitric oxide was verified with the Griess assay (Fig. S1B). After 6 h incubation cell culture supernatants (100 µl) were collected and mixed with equal volume (50 µL each) of Griess reagents A (5% phosphoric acid containing 1% sulfanilamide) and B (0.1% solution of N-(1-naphthyl)ethylenediamine in distilled water (dH_2O)); both from Sigma-Aldrich (Saint Louis, MO, USA). After 10 min at RT, the optical density (O.D.) was measured at 540 nm using an Infinite 200 Pro spectrophotometer.

Cytokine measurement in murine samples

In some experiments, cell supernatants were assayed for mouse IL-1β and IL-6 levels after 6 h of incubation using Mouse IL-1β ELISA Ready-SET-Go (Proteintech, Wuhan, China) and Mouse IL-6 ELISA Ready-SET-Go (Proteintech, Wuhan, China) (Fig. S7). The assays were performed according to the manufacturer's instructions.

Verification of LPS uptake by erythrocytes in neutrophil murine co-cultures – extended information

To determine whether the erythrocyte sequesters LPS in co-culture with PMN, we used AlexaFluor 568-conjugated LPS (Escherichia coli O55:B5; Invitrogen/ThermoFisher Scientific, Waltham, MA, USA). This allowed visualization of LPS localization and assessment of its cellular uptake within the co-culture. Erythrocytes and neutrophils were isolated as described above, counted, and adjusted to 2×10^6 and 5×10^5 cells, respectively, in 100 µL HBSS (+). Cells were incubated for 30 min at 37°C in 5% CO_2, then stimulated with fluorescent LPS (10 µg/mL) for 6 h under the same conditions. After incubation, erythrocytes were stained with anti-TER-119-AlexaFluor 647, and neutrophils with anti-Ly6G-Brilliant Violet 421 (both from BioLegend, San Diego, CA, USA), for 30 min at room temperature. Cell suspensions were transferred into cytospin chambers with glass slides, centrifuged twice (2500 × g, 6 min, 22°C), air-dried, and fixed with 4% PFA (10 min). Slides were counterstained with SYTOX Green (5 µM, for 5 min) and mounted in VECTASHIELD Mounting Medium (Vector Laboratories, Burlingame, CA, USA). Confocal imaging was performed using a ZEISS Axio Examiner.Z1 microscope. LPS-binding and/or LPS-internalizing cells were quantified at 40× magnification and expressed as the percentage of LPS-positive cells. In selected experiments, cells were pretreated for 30 min with neutralizing antibodies against TLR4/MD-2 (10 µg/mL, clone MTS510, Thermo Fisher Scientific, Waltham, MA, USA) or against CD14 (20 µg/mL, clone 4C1/CD14, BD Biosciences, San Jose, CA, USA). Control groups received isotype-matched antibodies: IgG2a (clone eBR2a, Thermo Fisher Scientific, Waltham, MA, USA) or IgG2b (clone A95-1, BD Biosciences, San Jose, CA, USA).

Murine or human inhibitors of Siglec receptors – extended information

In murine assays, neutrophils were preincubated with neutralizing antibodies targeting Siglec-E (0.35 µg/mL; Ultra-LEAF™, clone M1305A02, BioLegend, San Diego, CA, USA), Siglec-G (10 µg/mL; clone 805903, R&D Systems, Minneapolis, USA), or Siglec-F (20 µg/mL; clone 238023, R&D Systems, Minneapolis, USA). Matched isotype controls: rat IgG2a, κ (Ultra-LEAF™ IgG2a, κ, clone RTK2758; BioLegend, San Diego, CA, USA), rat IgG2a κ (clone 54447, R&D Systems, Minneapolis, USA), and rat IgG2b (clone 141945R, R&D Systems, Minneapolis, USA) were applied at corresponding concentrations in parallel control groups. In human studies, neutrophils were pretreated (30 minutes) with anti-human Siglec-9 (0,075 µg/mL; 191240, R&D Systems, Minneapolis, USA) neutralizing antibodies or isotype-matched IgG (mouse IgG2a κ isotype control antibody; 20102, R&D Systems, Minneapolis, USA) before RBCs and/or LPS stimulation.

***Ex vivo* blockade of CD24 on murine erythrocytes**

To neutralize CD24 on murine erythrocytes before their addition to co-cultures, cells were incubated for 1 h at 37°C and 5% CO_2 with a neutralizing anti-CD24 antibody (5 µg/mL; clone SN3, Santa Cruz Biotechnology, Dallas, TX, USA). Control erythrocytes were treated in parallel with an isotype-matched IgG1 κ antibody at the same concentration (clone 14G11, Santa Cruz Biotechnology Dallas, TX, USA). Following incubation, cells were centrifuged (1500 × g, 22°C, 5 min), resuspended in HBSS (+), and added to neutrophil cultures.

Characterization of Erythrocyte Surface *N*-Glycans by MALDI-ToF-MS – extended information

Glycoprotein Deglycosylation Procedure: For hemoglobin depletion, erythrocytes were incubated in ice-cold 5 mM sodium phosphate buffer (pH 7.4) for 30 min and centrifuged (12,000 rpm, 15 min, 4°C). Pellets were washed until a clear supernatant was obtained, yielding erythrocyte ghosts. Cells were lysed in RIPA buffer with protease inhibitors (ThermoFisher Scientific, Waltham, MA, USA) and proteins were precipitated using the methanol–chloroform method. Protein pellets were dried, dissolved in 1% SDS (Sigma-Aldrich), and denatured at 60°C for 10 min. For enzymatic *N*-glycan release, denatured proteins were supplemented with sodium phosphate buffer (50 mM final concentration, pH 7.5) and NP-40 (1% final concentration), (both from New England Biolabs, Ipswich, MA, USA), followed by incubation with PNGase F (100,000 U/mL; Promega, Madison, WI, USA) overnight at 37°C.

Linkage-Specific Derivatization and Purification of N-Glycans: Linkage-specific modification of sialic acids was performed to discriminate α2,3- and α2,6-linked residues by MALDI-TOF mass spectrometry. The approach follows the esterification/lactonization strategy described by Reiding et al. (3). In brief, to 2 µL of deglycosylation mixture, 20 µL of freshly prepared reaction buffer containing 0.25 M EDC (Pol-Aura, Poland) and 0.25 M HOBt (Acros Organics) in ethanol was added. Samples were incubated for 1 h at 37 °C. After derivatisation, 20 µL of acetonitrile was added, mixed thoroughly, and the mixtures were briefly centrifuged before purification or stored at −20 °C. In-house prepared cotton HILIC microtips were conditioned by washing three times with 15 µL Milli-Q water and equilibrated with three washes of 85% acetonitrile (AcN). Samples were loaded by aspirating/dispensing through the tips ten times. Bound glycans were washed sequentially with three volumes of 85% AcN containing 0.1% TFA and three volumes of 85% AcN. Elution was performed with 15 µL Milli-Q water.

MALDI-TOF-MS Analysis: Mass spectrometric measurements were carried out on a rapifleX MALDI-TOF system (Bruker Daltonics, Bremen, Germany) equipped with a Smartbeam 3D laser. Spectra were recorded in positive-ion reflectron mode using the following parameters: 5000 Hz laser repetition rate, 20 kV acceleration voltage, and a total accumulation of 32,000 laser shots per sample. Prior to analysis, the instrument was externally calibrated with Peptide Calibration Mix II (Bruker Daltonics). The m/z interval of 500–5000 was selected to appropriately cover the expected glycan species. For sample preparation, 1 µL of super-DHB matrix solution (5 mg/mL in 50% acetonitrile supplemented with 1 mM NaOH) was deposited onto an MTP AnchorChip 384 BC target plate (Bruker Daltonics). Subsequently, 1 µL of the HILIC-purified glycan sample was applied directly onto the matrix layer. Spots were allowed to dry at ambient conditions. To improve crystal uniformity, each spot was recrystallized by adding 0.5 µL of methanol.

MALDI-ToF-MS Data Analysis. Spectral pre-processing and peak detection were conducted using FlexAnalysis 4.2 (Bruker Daltonics). Assignment of *N*-glycan compositions was performed based on accurate mass using GlycoWorkbench platform (4). For quantitative analysis, the relative abundance of each glycan species was determined by normalizing its peak area to the total glycan signal for each spectrum. Glycosylation traits (sialylation) were subsequently derived from these normalized glycan abundances.

Immunocytochemical staining of murine or human cells – neutrophil extracellular trap (NET) formation – extended information

Following 6 h (murine neutrophils) or 3 h (human neutrophils) of incubation, cells were sequentially fixed with paraformaldehyde (PFA; ThermoScientific, Waltham, MA, USA) at increasing concentrations of 1% (2 min), 2% (10 min), and 3% (20 min), followed by washing twice with PBS. Subsequently, extracellular staining was performed to detect citrullinated histone H3 (citH3), neutrophil elastase (NE) and extracellular DNA (extDNA), For immunostaining, coverglasses were rinsed with PBS and then blocked with 3% bovine serum albumin (BSA, Sigma–Aldrich) in PBS for 45 minutes in a humid chamber at RT. Next, fixed cells were incubated with rabbit polyclonal anti-histone H3 (citrulline R2+R8+R17) antibodies (Abcam, Cambridge, UK) or rabbit polyclonal anti-NE antibodies (Cell Signaling Technology, Massachusetts, USA) diluted 1:200 in 1% BSA/PBS. The incubation with primary antibodies was carried out for 18 hours at 4°C in a humid chamber. Afterward, coverglasses were washed twice in PBS (5 min each) and incubated for one hour at room temperature in the dark with Cy3-conjugated goat anti-rabbit IgG (H+L) secondary antibodies

(1:300 in 1% BSA/PBS; Jackson ImmunoResearch Laboratories, Baltimore Pike, OH, USA). To visualize extracellular DNA, cells were stained with 5 µM SYTOX Green (ThermoFisher Scientific, Waltham, MA, USA) for 5 minutes, then washed with PBS. After completing the staining procedure, coverglasses were mounted onto slides using VECTASHIELD Mounting Medium (Vector Laboratories, Burlingame, CA, USA).

Immunocytochemical staining of Siglec receptor expression on murine or human neutrophils – extended information

Immunostaining of Siglec-E/-G/-F (murine cells) and Siglec-9 (human cells) receptor expression was performed. After 6 h (murine) or 3 h (human) of incubation, cells were fixed with PFA as described above, and slides were blocked with 3% BSA/PBS. Subsequently, 2 h incubation with rat monoclonal anti-Siglec-E antibody conjugated with PE (clone M1304A01; BioLegend, San Diego, CA, USA) and/or with rat monoclonal anti-Siglec-G antibody conjugated with Brilliant Violet 421 (clone SH1; BioLegend, San Diego, CA, USA) or rat monoclonal anti-Siglec-F conjugated with AlexaFluor 488 (clone S17007L; BioLegend, San Diego, CA, USA) diluted 1:50, 1:20 and 1:50 respectively in 1% BSA/PBS was performed (4°C, in a humid chamber). As for human neutrophils, after blocking with 3% BSA/PBS for 45 minutes, the slides were incubated for 2 hours with a mouse monoclonal anti-human Siglec-9 antibody conjugated with Alexa Fluor 647 (R&D Systems, Minneapolis, USA) diluted 1:50 in 1% BSA/PBS. Next, the coverslips were washed twice in PBS, then stained with SYTOX green (5 µM) or Hoechst 33342 (10 µM; both from ThermoFisher Scientific, Waltham, MA, USA) for 5 minutes at RT. Slides were washed in PBS and mounted with VECTASHIELD medium.

Immunocytochemical staining of SHP-1 and Siglec-E localization

In selected experiments, downstream inhibitory signaling was assessed by analyzing proximal localization of Siglec-E with SHP-1 at the surface/in the cell membrane of intact and endotoxemic PMNs. Immunocytochemistry was performed as follows. Cells were permeabilized with 0.1% Triton X-100 in PBS for 5 min and blocked with 3% BSA in PBS for 45 min. Slides were then incubated overnight at 4°C with rabbit monoclonal anti-SHP-1 antibody (1:50 in 1% BSA/PBS; Cell Signaling Technology, USA). After washing in PBS, samples were incubated for 2 h at room temperature with goat anti-rabbit IgG (Cy5) secondary antibody (1:300 in 1% BSA/PBS; Jackson ImmunoResearch Laboratories, USA) and rat monoclonal anti-Siglec-E antibody conjugated with PE (1:50 in 1% BSA/PBS; clone M1304A01; BioLegend, USA). Next, the coverslips were washed twice in PBS, then stained with SYTOX green (5 µM) for 5 minutes at RT. Slides were washed in PBS and mounted with VECTASHIELD medium.

Detection of murine RBC- PMN interactions – extended information

In order to detect and visualize isolated RBCs, cells were counted, resuspended in HBSS (+) and brought to a density of 2 × 10^5/200 µL cells per Eppendorf tube. Cells were labeled with PE anti-mouse TER-119 antibody (clone TER-119, BioLegend, San Diego, CA, USA) for 2 h at 37°C, 5% CO_2. Following incubation, the samples were centrifuged (1,500 rcf, 22°C, 5 min), and the resulting pellet was gently resuspended in HBSS (+). The cells were then layered onto a microscope slide and imaged with a ZEISS Axio Examiner.Z1 upright microscope equipped with a metal halide light source (AMH-200-F6S; Andor, Oxford Instruments) and with a DSD2 spinning-disk confocal module (Andor, Oxford Instruments). In order to identify RBC-PMN interactions after their co-incubation, cells were resuspended in HBSS (+) in a 4:1 ratio and labelled (4 h at 37°C, 5% $CO_{2)}$ with PE anti-mouse TER-119 antibody (clone TER-119, BioLegend, San Diego, CA, USA) and Brilliant Violet 421 anti-mouse Ly6G antibody (clone 1A8, BioLegend, San Diego, CA, USA) respectively. After incubation, cells were centrifuged (1,500 rcf, 22°C, 5 min), and the pellet was resuspended in HBSS (+). Subsequently, cell suspensions were applied onto glass coverslips and examined by confocal microscopy to visualize erythrocyte-neutrophil interactions.

Microscopic analysis

Following immunocytochemical staining, fluorescence imaging was performed using a ZEISS Axio Examiner.Z1 upright microscope equipped with a spinning-disk device DSD2 (with ZEISS EC Plan-NEOFLUAR 20 × /0.5 and ZEISS LD Plan-NEOFLUAR 40 × /0.6) and a metal halide light source

(AMH-200-F6S; Andor, Oxford Instruments, Abingdon, UK). Fluorescent signal was detected by 5.5 megapixel sCMOS camera (Zyla 5.5, Andor Oxford Instruments, Abingdon, UK). NET formation, Siglec receptor expression and SHP-1 localization were analyzed in defined fluorescence channels. For NET formation analysis imaging was performed in two fluorescence channels: GFP (green fluorescent protein; excitation 482/18 nm, emission 525/45 nm) and RFP (red fluorescent protein; excitation 561/54 nm, emission 609/54 nm) in utilizing IQ Live Cell Imaging software (Andor, Oxford Instruments, Abingdon, UK). Acquired images were analyzed using ImageJ v1.53a software (National Institutes of Health, USA). Fluorescent images were converted to 8-bit grayscale, and the positive signal was quantified using the threshold function. For murine Siglecs expression immunostaining, imaging was performed using three fluorescence channels: Siglec-E expression was assessed with the RFP channel, while extDNA was detected in the GFP channel, Siglec-G expression was assessed with the DAPI channel (excitation: 390/40 nm, emission: 452/45 nm) while extDNA was detected in the GFP channel and Siglec-F expression was assessed with the GFP channel, while extDNA was detected in the DAPI channel. For Siglec-9 analysis, extDNA was visualized using the GFP channel, whereas Siglec-9 expression was captured in the CY5 channel (excitation: 640/14 nm, emission: 676/29 nm). In all cases thresholded signals from the GFP/DAPI channel (representing extracellular DNA) and the RFP/DAPI/GFP/Cy5 channel (corresponding to Siglecs expression) were measured. The relative receptor expression was determined by calculating the RFP/GFP (Siglec-E), DAPI/GFP (Siglec-G), GFP/DAPI (Siglec-F), or CY5/GFP (Siglec-9) ratio [%], providing a normalized measure of receptor expression levels in relation to neutrophil number. To analyze the redistribution of SHP-1 to the cell membrane in a proximity of Siglec-E receptor, SHP-1 was visualized using the CY5 channel, whereas Siglec-E expression was captured in the RFP channel. The relative proximal localization of SHP-1 with Siglec-E was determined on Siglec-E-positive neutrophils by calculating the ratio [%] of cells showing membrane-associated SHP-1/Siglec-E overlap relative to the total number of Siglec-E-positive neutrophils. 3D models (series of optical sections, z-stacks) of Siglec-E/SHP-1 overlap on the cell surface/in the cell membrane were made.

Figures

Suppl Fig. 1

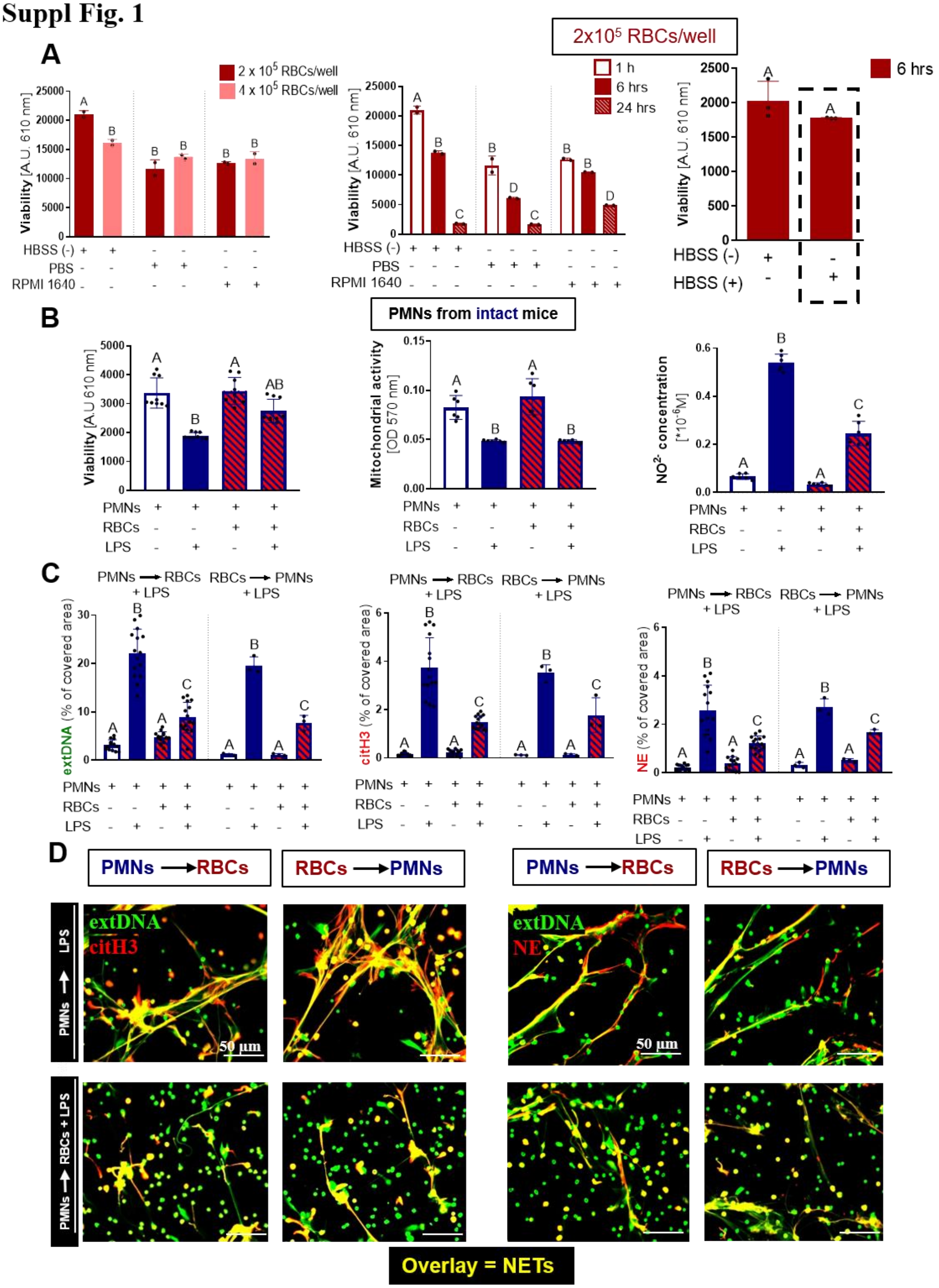

Fig. S1. Establishing culturing conditions of erythrocytes and their co-cultures with neutrophils, and effect of LPS timing on NET formation. Erythrocytes and neutrophils were isolated from whole blood and bone marrow of untreated (intact) C57BL/6J mice, respectively. To optimize conditions for maintaining erythrocytes in co-culture with neutrophils, (A) viability of the isolated RBCs at various concentrations (200 vs. 400 × 10^5 RBCs/well), culture media types (HBSS -/+, PBS, RPMI 1640) and incubation time (1, 6, 24 hrs) was tested. (B-D) Isolated cells were placed in either RBC:PMN co-cultures (4:1 ratio) or separate monocultures and then were stimulated with LPS (50 µg/ml) for 6 hours. (B) Neutrophil viability, mitochondrial activity, and nitric oxide (NO_2^-) production were assessed in the presence of RBCs and/or LPS. (C) NET formation depending on the order of cell seeding (neutrophils first or RBCs first) was estimated by application of antibodies detecting citrullinated histones 3 (citH3) or neutrophil elastase (NE) overlapping with the NET backbone, extracellular DNA (extDNA). Quantitative data shows the percentage of area occupied by individual NET components (extDNA, citH3, NE) released by neutrophils. (D) Representative images of NET formation (overlay; yellow) acquired at 20× optical magnification; scale bar indicates 50 µm. In graphs with RBCs viability (A) the black frame indicates a culture conditions selected for the majority of subsequent studies: RBCs cultured in HBSS (+) for 6 hours in the RBC:PMN ratio of 4:1. Results are presented as mean ± SD from at least three fields of view. Statistically significant differences were marked with letter symbols according to one-way ANOVA (Bonferroni post hoc); $n \geq 3$; A.U. - arbitrary units, HBSS (-) - Hanks' Balanced Salt Solution without Ca^{2+} and Mg^{2+}, HBSS (+) - Hanks' Balanced Salt Solution with Ca^{2+} and Mg^{2+}, PMN – neutrophils, RBC – erythrocytes.

Suppl Fig. 2

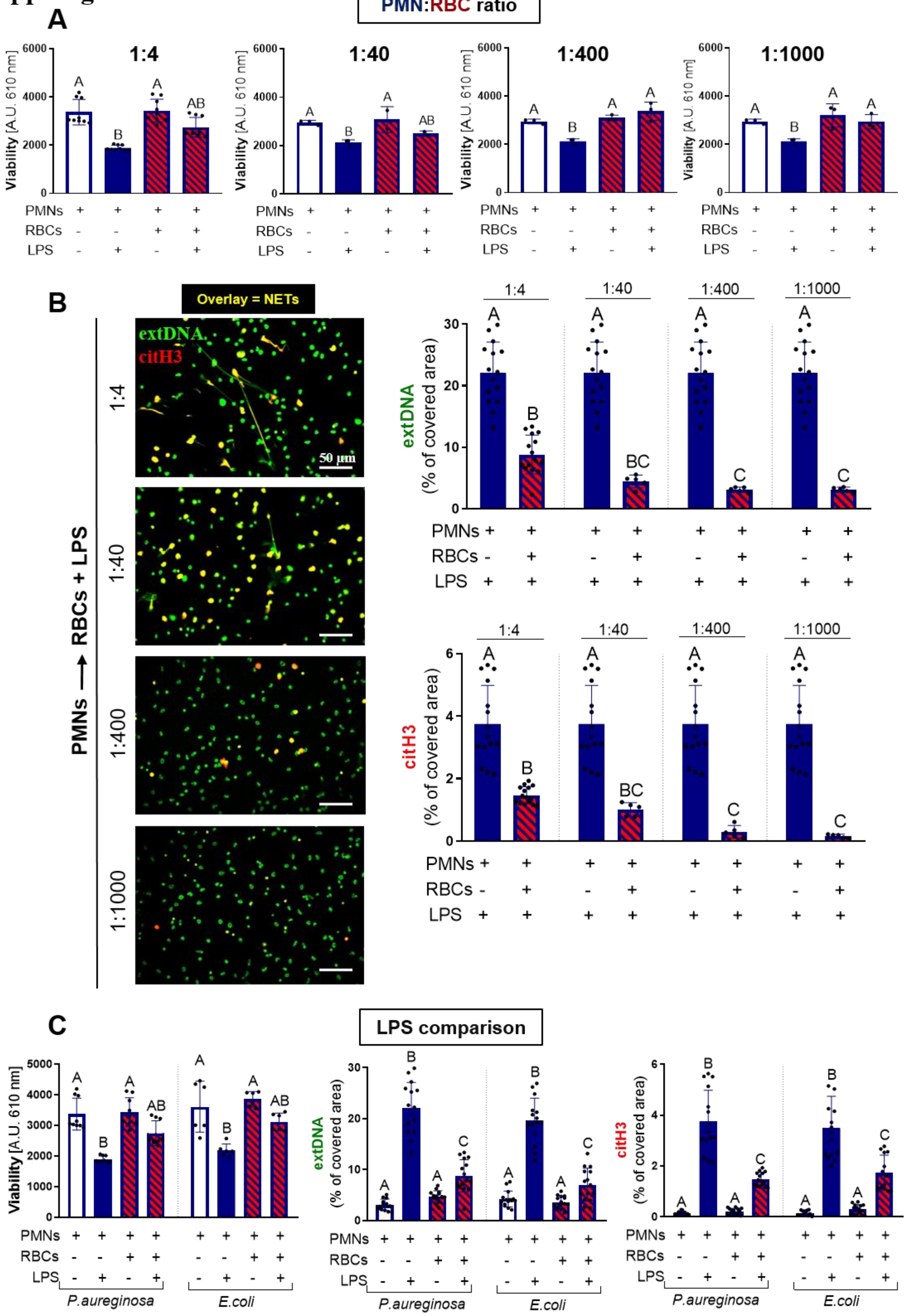
A
PMN:RBC ratio
1:4
1:40
1:400
1:1000
Viability [A.U. 610 nm]
PMNs
RBCs
LPS
B
Overlay = NETs
extDNA
citH3
50 µm
PMNs ⟶ RBCs + LPS
extDNA (% of covered area)
citH3 (% of covered area)
C
LPS comparison
P.aureginosa
E.coli

Fig. S2. Impact of ratio of erythrocytes and neutrophils on their viability and NET formation, and significance of the LPS source. Erythrocytes and neutrophils were isolated from whole blood and bone marrow of untreated (intact) C57BL/6J mice, respectively. Isolated cells were placed in PMN:RBC co-cultures in various cell ratios (1:4, 1:40, 1:100, 1:1000) and (A) viability and (B) NET formation was assessed. Cells were co-cultured for 6 hrs, with or without LPS (50 µg/ml). (B) NET formation depending on the cell ratio was estimated by application of antibodies detecting citrullinated histones 3 (citH3, red) overlapping extracellular DNA (extDNA, green). Representative fluorescence images (left) show overlaid signals from both components (yellow) that marks NET structures; scale bar indicates 50 µm. Quantitative data (right) shows the percentage of area occupied by individual NET components (extDNA, citH3) released by neutrophils. (C) Neutrophils were cultured in the presence of RBCs and/or LPS (50 µg/ml) derived from *Pseudomonas aeruginosa* or *Escherichia coli* (75 µg/ml, as indicated) for 6 hrs prior to viability and NET evaluation. Results are presented as mean ± SD from at least three fields of view. Statistically significant differences were marked with letter symbols according to one-way ANOVA (Bonferroni post hoc); n ≥ 3; PMN – neutrophils, RBC – erythrocytes.

Suppl Fig. 3

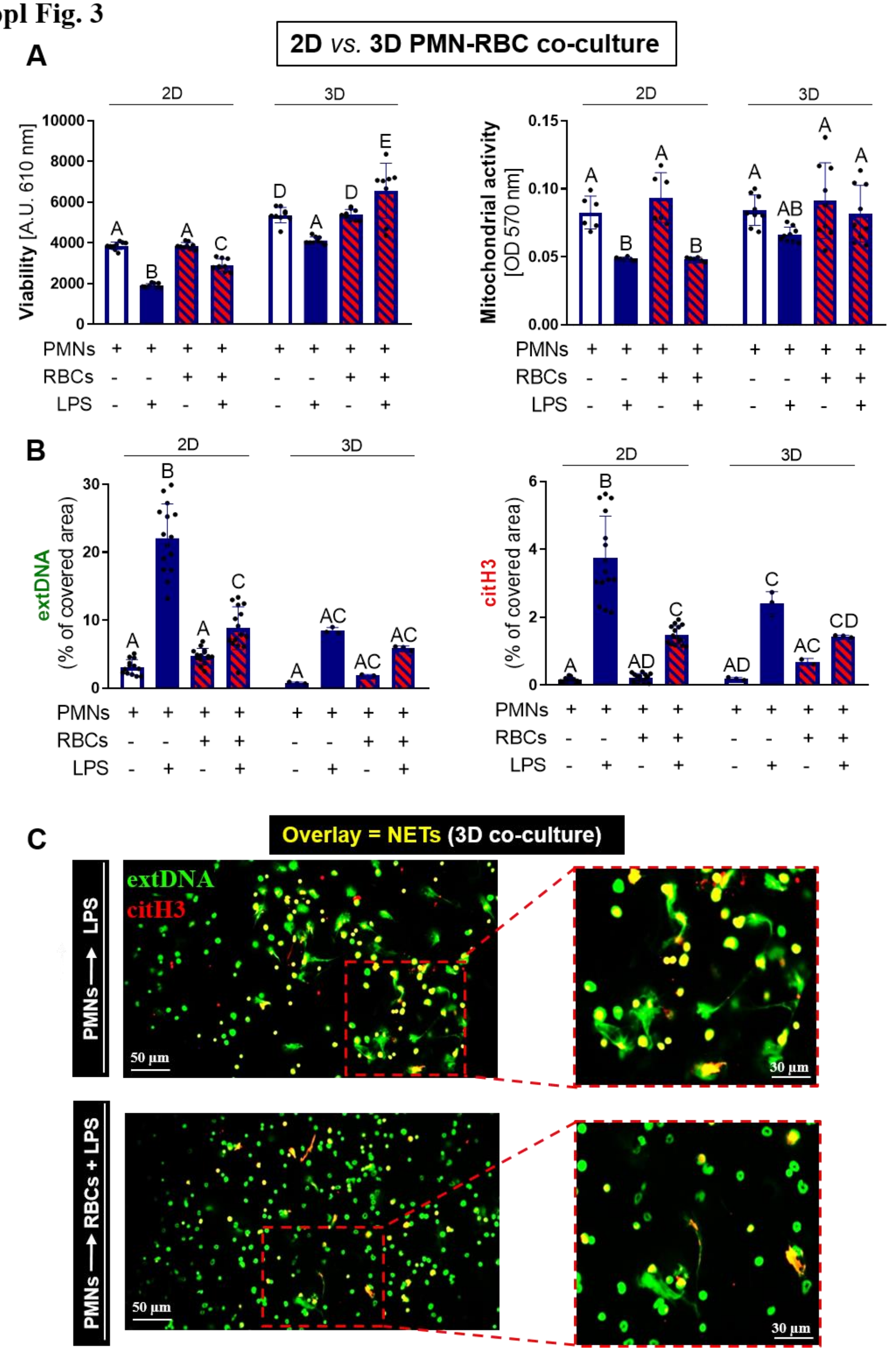
2D vs. 3D PMN-RBC co-culture
A
2D
3D
Viability [A.U. 610 nm]
Mitochondrial activity [OD 570 nm]
PMNs
RBCs
LPS
B
extDNA (% of covered area)
citH3 (% of covered area)
C
Overlay = NETs (3D co-culture)
extDNA
citH3
PMNs → LPS
PMNs → RBCs + LPS
50 µm
30 µm

Fig. S3. Capability of erythrocytes to inhibit NET formation in 2D versus 3D conditions. Erythrocytes and neutrophils were isolated from healthy C57BL/6J mice, from whole blood and bone marrow, respectively. Isolated cells were cultured either as monocultures (PMN only) or in 2D and 3D co-cultures with RBCs (based on the hydrogel PuraMatrix) at a PMN:RBC ratio of 1:4 and subsequently stimulated with LPS (50 µg/mL) for 6 h. Changes in (A) cell viability and mitochondrial activity and (B) NET levels were compared between different co-culture conditions (2D vs. 3D). NET levels were quantified based on the percentage of area occupied by individual NET components (extDNA and citrullinated histone H3) using ImageJ. (C) Representative images of NET formation in the presence of RBCs and/or LPS in 3D co-cultures. Visualization: extracellular DNA (extDNA, green), detected using the fluorescent DNA dye SYTOX Green; citrullinated histone H3 (citH3, red), detected using polyclonal primary anti-citH3 antibodies and Cy3-conjugated polyclonal secondary antibodies. Images from individual channels were merged to visualize co-localization of NET components. Images were acquired at 20× optical magnification; scale bars indicate 30 or 50 µm, as labeled in the images. Results are presented as mean ± SD from at least three fields of view. Statistically significant differences were marked with letter symbols according to one-way ANOVA (Bonferroni post hoc); n ≥ 3; PMN – neutrophils, RBC – erythrocytes.

Suppl Fig. 4

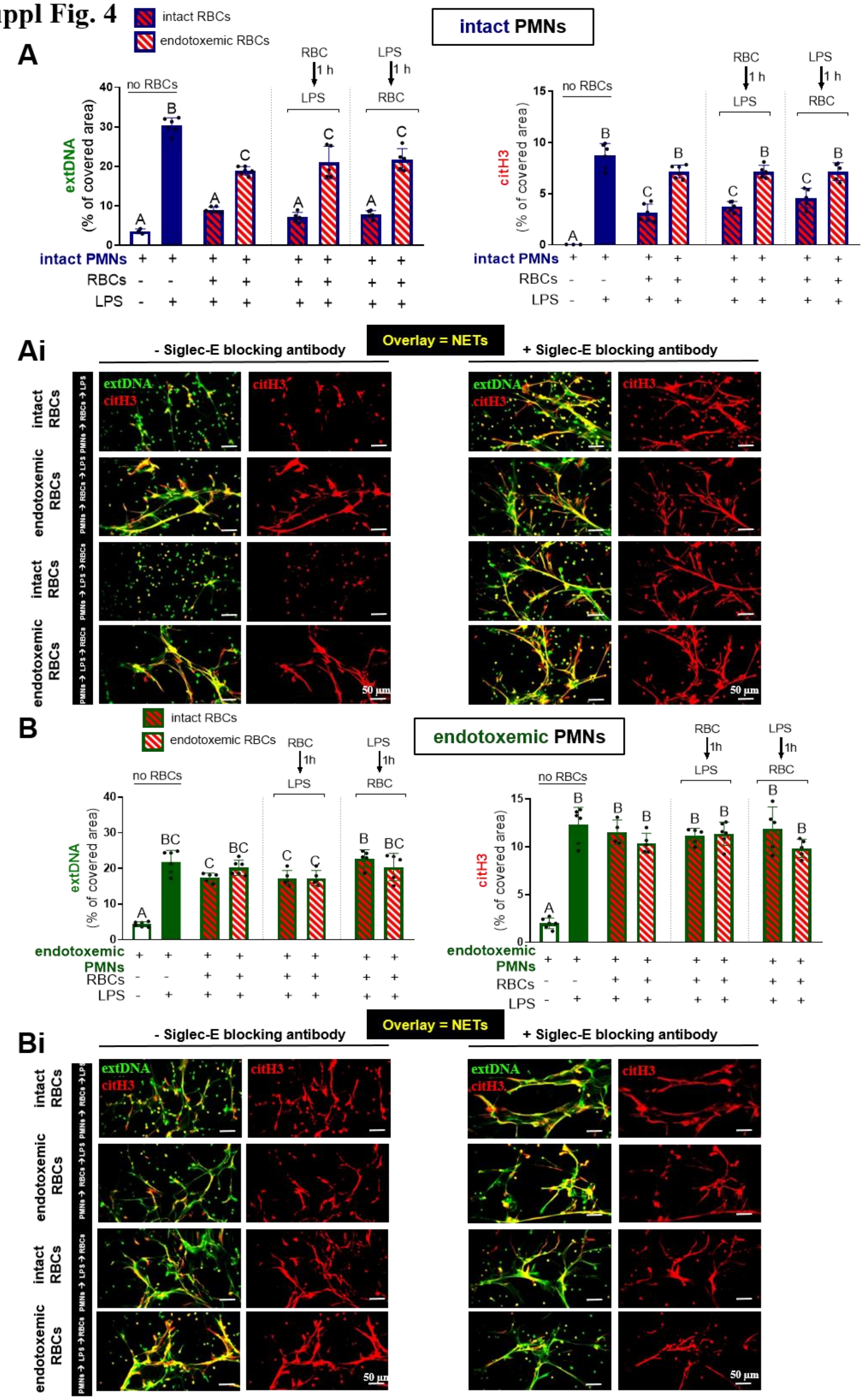

A
intact RBCs
endotoxemic RBCs
intact PMNs
no RBCs
RBC
1 h
LPS
extDNA
(% of covered area)
citH3
intact PMNs
RBCs
LPS
Ai
- Siglec-E blocking antibody
Overlay = NETs
+ Siglec-E blocking antibody
extDNA
citH3
intact RBCs
endotoxemic RBCs
50 µm
B
endotoxemic PMNs
1h
endotoxemic PMNs
Bi

Fig. S4. Effect of timing of erythrocyte addition to neutrophil cultures on their capacity to inhibit NETs in relation to Siglec-E expression. Erythrocytes and neutrophils were isolated from whole blood and bone marrow of healthy and endotoxemic C57BL/6J mice at early phase of inflammation (8 hours; i.p. LPS, 1 mg/kg body weight). The cells were cultured with or without mixing (e.g. healthy PMNs with endotoxemic RBCs) and upon LPS stimulation (50 µg/ml, 6 hrs). Isolated RBCs were added to co-cultures either simultaneously with LPS,1 h before LPS addition, or 1 h after LPS addition, to compare the effects of different stimulation strategies on NET release by (A-Ai) intact or (B-Bi) endotoxemic PMNs. NET formation was estimated by application of antibodies detecting citrullinated histones 3 (citH3, red) and extracellular DNA staining with SYTOX Green (extDNA, green). (A, B) Quantitative data shows the percentage of area occupied by individual NET components (extDNA, citH3) released by neutrophils. (Ai, Bi) Representative images of NET formation (overlay; yellow), images were acquired at 20× optical magnification; scale bar indicates 50 µm. Results are presented as mean ± SD from at least three fields of view. Statistically significant differences were marked with letter symbols according to one-way ANOVA (Bonferroni post hoc); n ≥ 3; PMN – neutrophils, RBC – erythrocytes.

Suppl Fig. 5

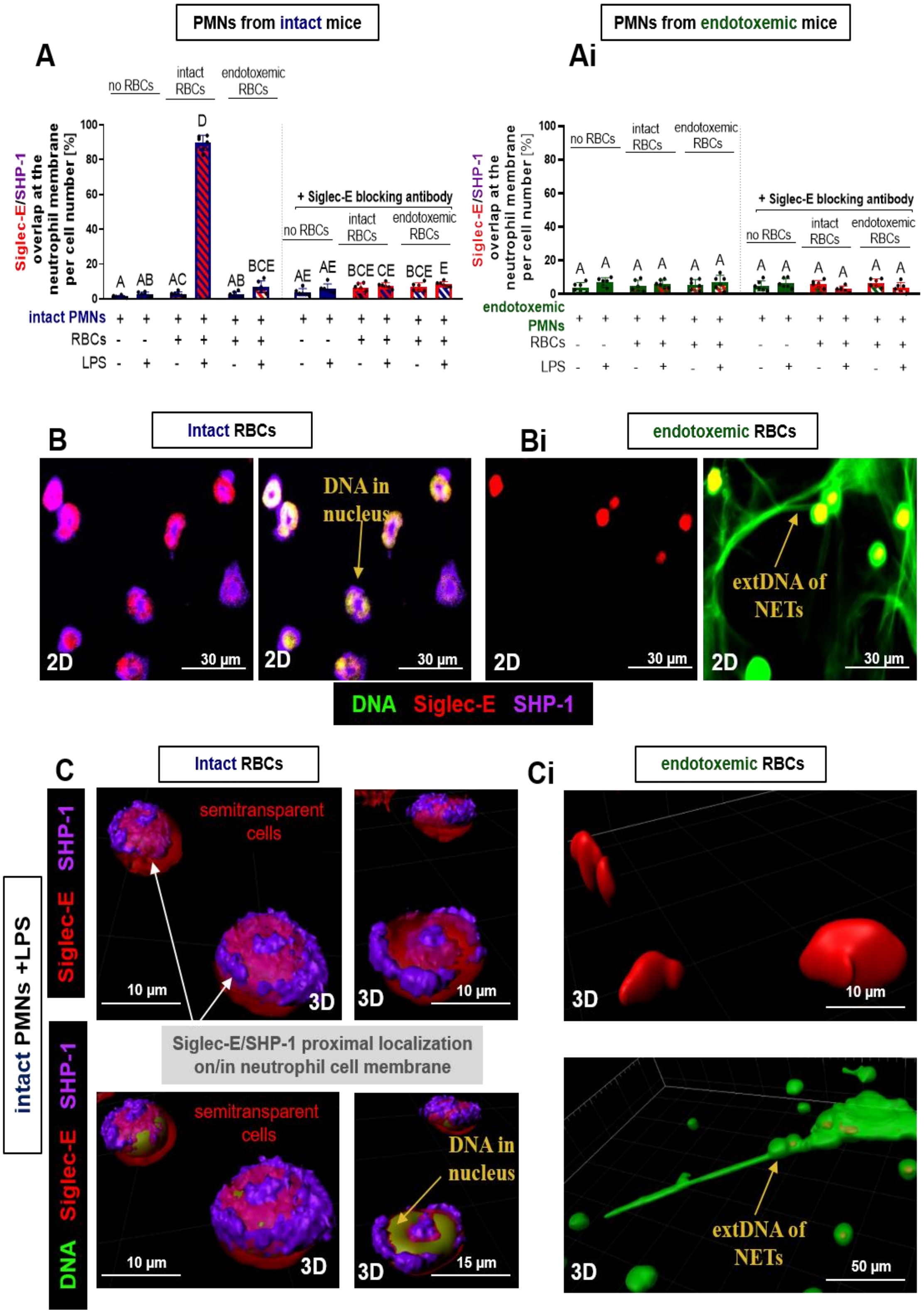
PMNs from intact mice
PMNs from endotoxemic mice
A
Ai
Siglec-E/SHP-1 overlap at the neutrophil membrane per cell number [%]
no RBCs
intact RBCs
endotoxemic RBCs
+ Siglec-E blocking antibody
intact PMNs
endotoxemic PMNs
RBCs
LPS
B
Bi
Intact RBCs
endotoxemic RBCs
DNA in nucleus
extDNA of NETs
2D
30 µm
DNA Siglec-E SHP-1
C
Ci
intact PMNs +LPS
Siglec-E SHP-1
DNA Siglec-E SHP-1
semitransparent cells
Siglec-E/SHP-1 proximal localization on/in neutrophil cell membrane
3D
10 µm
15 µm
50 µm

Fig. S5. In activated neutrophils co-cultured with erythrocytes, SHP-1 is recruited to the cell membrane in a proximity of Siglec-E, but only in cells from intact mice. (A-Ai) Neutrophils isolated from bone marrow of intact or endotoxemic C57BL/6J mice were co-cultured in monocultures or in mixed co-cultures with intact or endotoxemic erythrocytes and were stimulated with LPS (50 µg/ml) for 6 h. Studies were also performed under conditions in which Siglec-E was blocked with anti-Siglec-E antibodies (0.35 µg/ml) 30 min prior to the experiments. The redistribution of SHP-1 to the cell membrane in a proximity of Siglec-E receptor on the surface of neutrophils form intact (A) and endotoxemic (Ai) mice were analyzed with ImageJ, and are expressed as % of cells per cell number. Representative 2D images (B-Bi) and 3D models (z-stacks; C-Ci) of Siglec-E/SHP-1 proximal localization on the cell surface/in the cell membrane are shown. Neutrophils were stained with monoclonal antibodies against Siglec-E (red) and primary monoclonal anti-SHP-1 antibodies, followed by Cy5-conjugated polyclonal secondary antibodies (purple). Cell nuclei/extDNA were counterstained with SYTOX green (as indicated in selected images). The Siglec-E signal was made semitransparent to visualize the overlap with SHP-1. Images were acquired at 40× optical magnification; scale bar indicates 10, 15, 30 or 50 µm, as labeled in the images. Results are presented as mean ± SD. Statistically significant differences were marked with letter symbols according to one-way ANOVA (Bonferroni post hoc); n ≥ 3. extDNA – extracellular DNA, PMN – neutrophils, RBC – erythrocytes.

Suppl Fig. 6

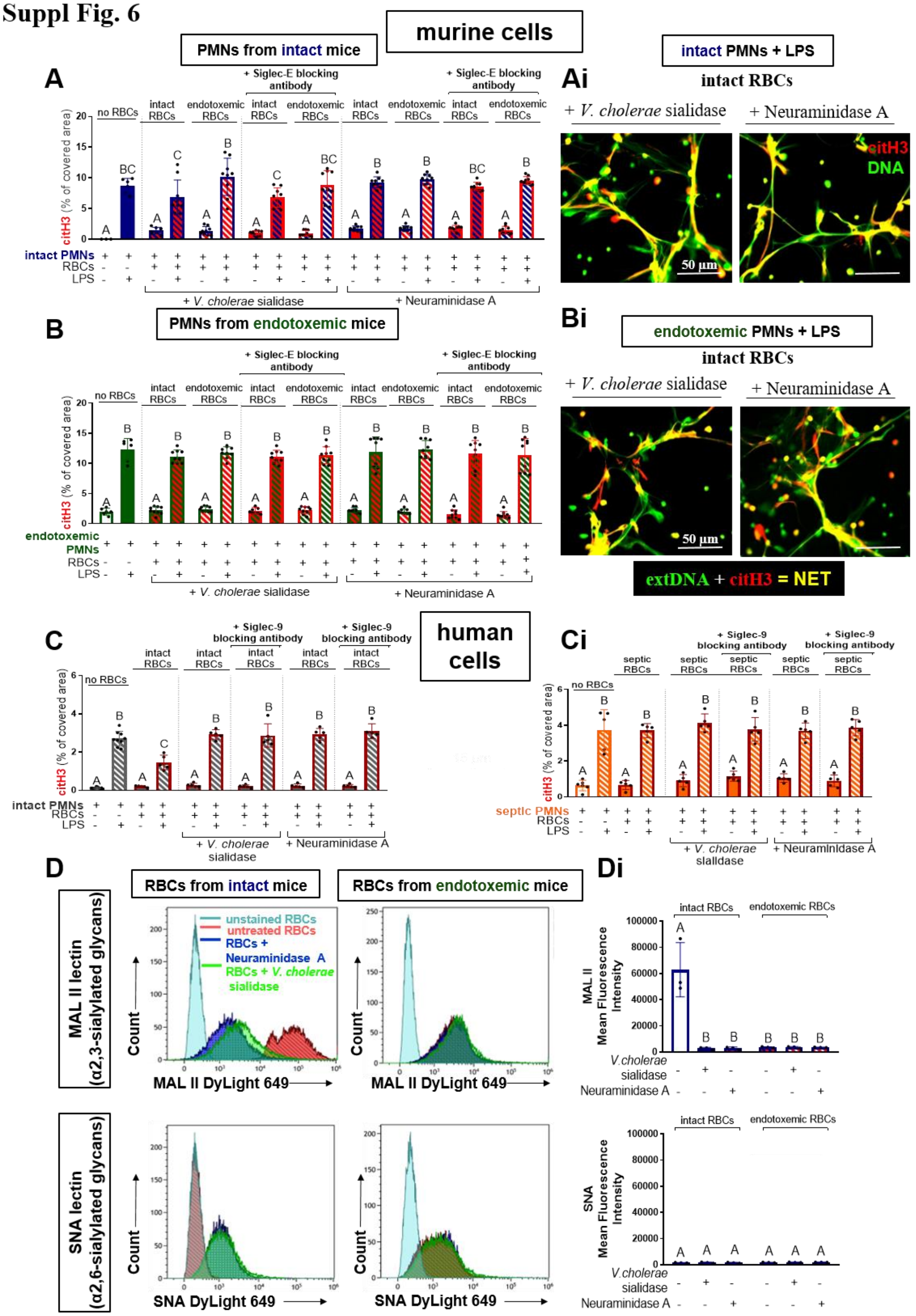

murine cells
A
PMNs from intact mice
citH3 (% of covered area)
intact PMNs
RBCs
LPS
+ V. cholerae sialidase
+ Neuraminidase A
+ Siglec-E blocking antibody
Ai
intact PMNs + LPS
intact RBCs
+ V. cholerae sialidase
+ Neuraminidase A
citH3
DNA
50 µm
B
PMNs from endotoxemic mice
endotoxemic PMNs
Bi
endotoxemic PMNs + LPS
extDNA + citH3 = NET
human cells
C
Ci
+ Siglec-9 blocking antibody
septic PMNs
septic RBCs
D
RBCs from intact mice
RBCs from endotoxemic mice
MAL II lectin (α2,3-sialylated glycans)
unstained RBCs
untreated RBCs
RBCs + Neuraminidase A
RBCs + V. cholerae sialidase
Count
MAL II DyLight 649
SNA lectin (α2,6-sialylated glycans)
SNA DyLight 649
Di
MAL II Mean Fluorescence Intensity
SNA Mean Fluorescence Intensity
intact RBCs
endotoxemic RBCs
V.cholerae sialidase
Neuraminidase A

Fig. S6. Erythrocyte desialylation disrupts NET inhibition in murine and human neutrophils and intact erythrocytes carry mostly α2,3-sialylated glycans. Erythrocytes and neutrophils were isolated from whole blood and bone marrow of intact or endotoxemic (8 h) C57BL/6J mice or from healthy donors and septic patients. Isolated cells were placed in PMN:RBC co-cultures with intact or endotoxemic/septic RBCs or in separate monocultures and then were stimulated with LPS (50 μg/ml) for 6 hours (mice cells) or 3 h (human cells). Studies were also performed under conditions in which Siglec-E/-9 was blocked with anti-Siglec-E/-9 antibodies (0.35 μg/ml and 0,075 μg/mL, respectively) 30 min prior to the experiments. (A-Ci) Changes in NET formation by murine (A, Ai – intact cells, B, Bi - endotoxemic cells) and human neutrophils (C – intact cells, Ci – cells from septic patients) depending on the level of RBCs sialylation. Neutrophils were co-cultured with erythrocytes that were either untreated or pre-treated (1 h) with *V. cholerae* sialidase (0.125 U) or broad-spectrum neuraminidase A (100 U) prior to analysis. (A-Ci) NET levels were quantified based on the percentage of area occupied by citrullinated histone H3 (citH3) using ImageJ. Representative images of NET formation by (Ai) intact PMNs and (Bi) endotoxemic PMNs in the presence of intact RBCs and LPS after *V. cholerae* sialidase or neuraminidase A treatment. Visualization: extracellular DNA (extDNA, green), detected using the fluorescent DNA dye SYTOX green; citrullinated histone H3 (citH3, red), detected using polyclonal primary anti-citH3 antibodies and Cy3-conjugated polyclonal secondary antibodies. Images from individual channels were merged to visualize the co-localization of NET components. Images were acquired at 20× optical magnification; scale bars indicate 50 μm. (D-Di) Erythrocyte surface sialylation was analyzed using lectin-based flow cytometry. RBCs isolated from intact or endotoxemic mice were stained with biotinylated-MAL II or SNA (both 5 μg/ml) lectins for 1 h, followed by incubation with DyLight 649-conjugated streptavidin (1 μg/ml, 1 h) and evaluated with flow cytometry. In selected experiments, RBCs were treated with *V. cholerae* sialidase (0.125 U) or broad-spectrum neuraminidase A (100 U) for 1 h prior to analysis. Representative histograms (D) and quantitative analysis of lectin binding, expressed as mean fluorescence intensity (Di), are shown. Results are presented as mean ± SD. Statistically significant differences were marked with letter symbols according to one-way ANOVA (Bonferroni post hoc); $n \geq 3$. extDNA – extracellular DNA, MAL II - Maackia Amurensis Lectin II, PMN – neutrophils, RBC – erythrocytes, SNA - Sambucus Nigra Lectin.

Suppl Fig. 7

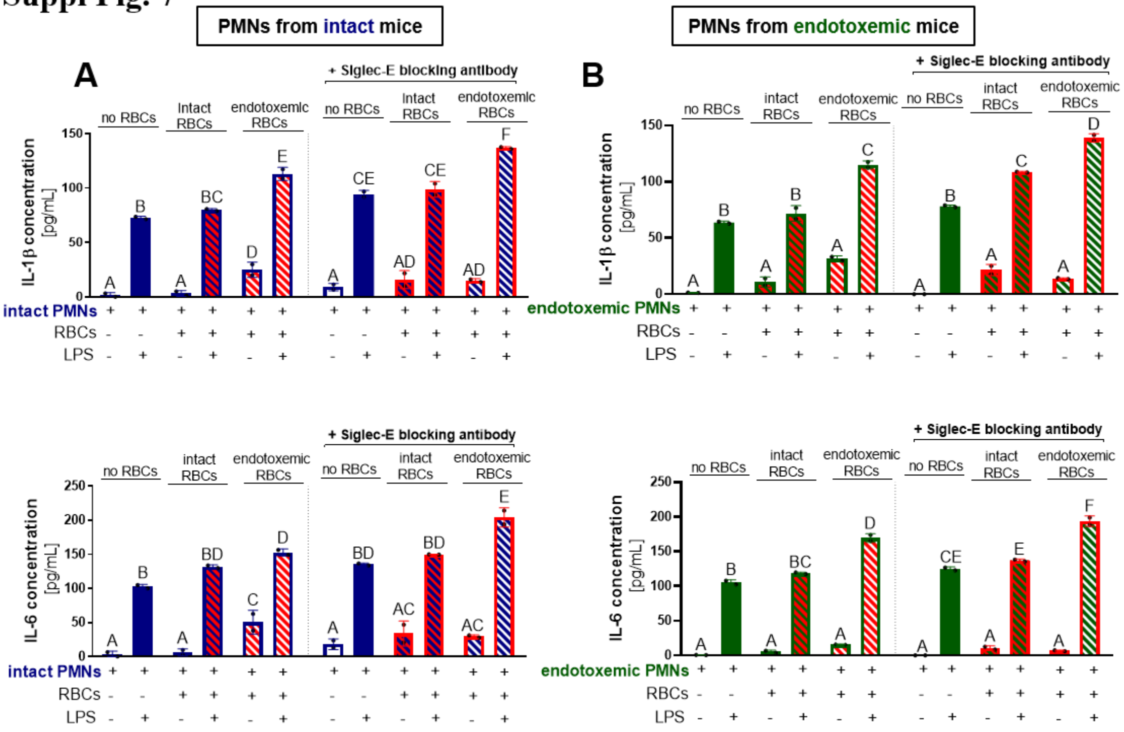

Fig. S7. Erythrocytes inhibit pro-inflammatory cytokine release by neutrophils. Erythrocytes and neutrophils were isolated from healthy and endotoxemic C57BL/6J mice (8 h after i.p. administration of LPS, 1 mg/kg body weight). Cells were cultured as monocultures (healthy or endotoxemic PMN) or in co-culture with RBCs at a PMN:RBC ratio of 1:4, in homogeneous combinations (healthy or endotoxemic cells only) or mixed combinations (healthy PMN with endotoxemic RBCs or endotoxemic PMN with healthy RBCs), and then incubated for 6 h in the presence of LPS (50 µg/mL). In selected experiments, monoclonal blocking antibodies against Siglec-E (0.35 µg/mL) were added to the cells 30 min before the addition of RBCs and/or LPS. Production of IL-1β and IL-6 in (A) intact and (B) endotoxemic PMN culture supernatants was quantified by ELISA. Results are presented as mean ± SD. Experiments were performed in three independent replicates. Statistically significant differences were indicated with letter symbols according to one-way ANOVA (Bonferroni post hoc); n ≥ 3; IL-1β - interleukin-1β, IL-6 - interleukin-6, PMN – neutrophils, RBC – erythrocytes.

Suppl Fig. 8

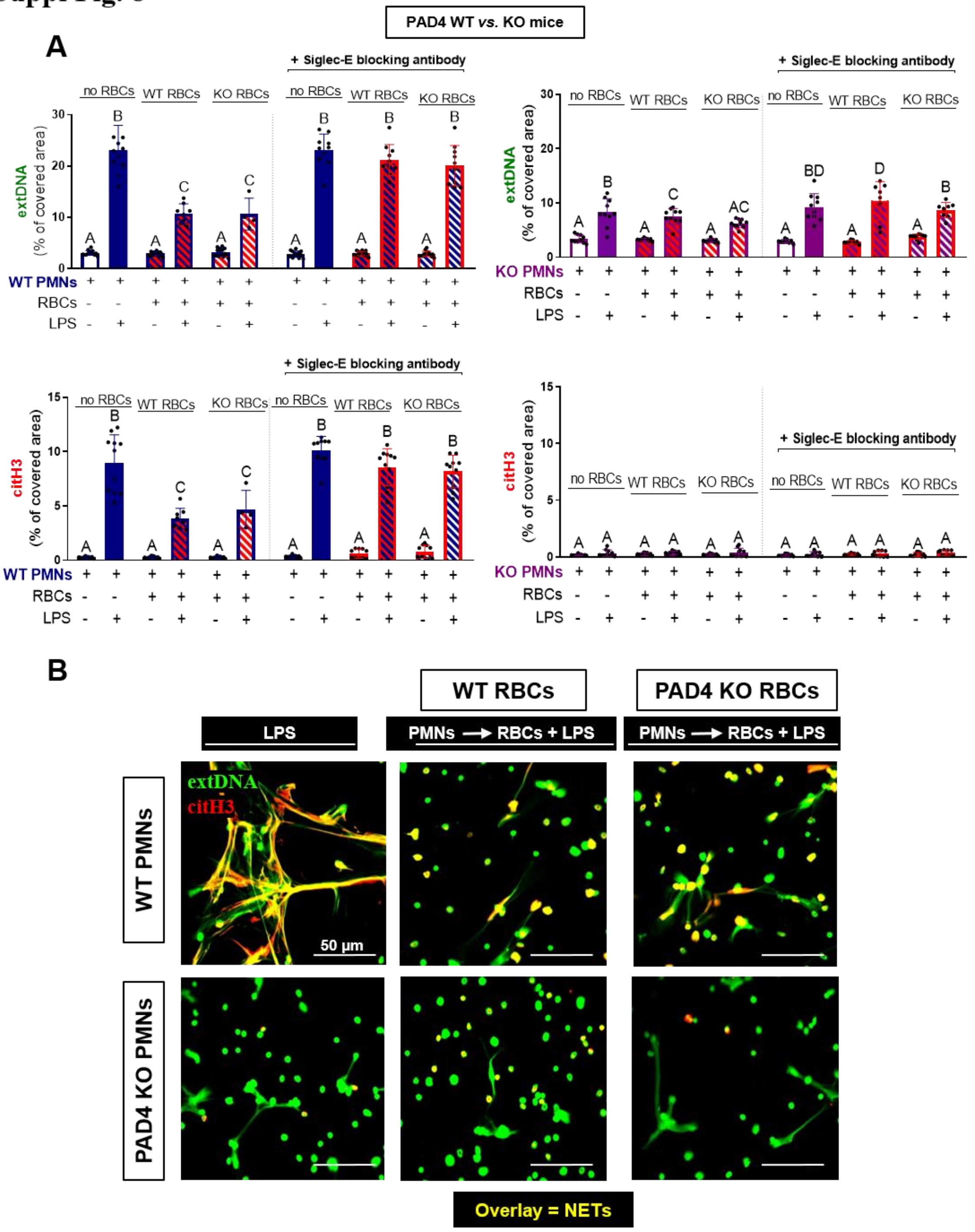
PAD4 WT vs. KO mice
A
+ Siglec-E blocking antibody
no RBCs
WT RBCs
KO RBCs
extDNA
(% of covered area)
citH3
WT PMNs
KO PMNs
RBCs
LPS
B
WT RBCs
PAD4 KO RBCs
LPS
PMNs → RBCs + LPS
extDNA
citH3
50 µm
WT PMNs
PAD4 KO PMNs
Overlay = NETs

Fig. S8. Impact of Peptidylarginine Deiminase 4 (PAD4) on Siglec-E-dependent inhibition of NET formation by erythrocytes. Erythrocytes and neutrophils were isolated from intact wild-type (WT) C57BL/6J mice or PAD4 knockout ($PAD4^{-}/^{-}$; KO) mice. Cells were cultured as monocultures (WT or KO PMNs) or in co-culture with RBCs at a PMN:RBC ratio of 1:4, in homogeneous combinations (WT or KO cells only) or mixed combinations (WT PMNs with KO RBCs or KO PMNs with WT RBCs), and then incubated for 6 h in the presence of LPS (50 μg/mL). (A) Quantitative data showing the percentage of area occupied by individual NET components (extracellular DNA and citrullinated histone H3) released by neutrophils, as determined by ImageJ analysis. (B) Representative images of NET formation in the presence of RBCs and/or LPS, depending on the combinations of cell types used in co-cultures. Visualization: extracellular DNA (extDNA, green), detected using the fluorescent DNA dye SYTOX Green; citrullinated histone H3 (citH3, red), detected using polyclonal primary anti-citH3 antibodies and Cy3-conjugated polyclonal secondary antibodies. Images from individual channels were merged to visualize co-localization of NET components. Images were acquired at 20× optical magnification; the scale bar indicates 50 μm. Results are presented as mean ± SD from at least three fields of view. Statistically significant differences were marked with letter symbols according to one-way ANOVA (Bonferroni post hoc); $n \geq 3$; PMN – neutrophils, RBC – erythrocytes.

Suppl Fig. 9

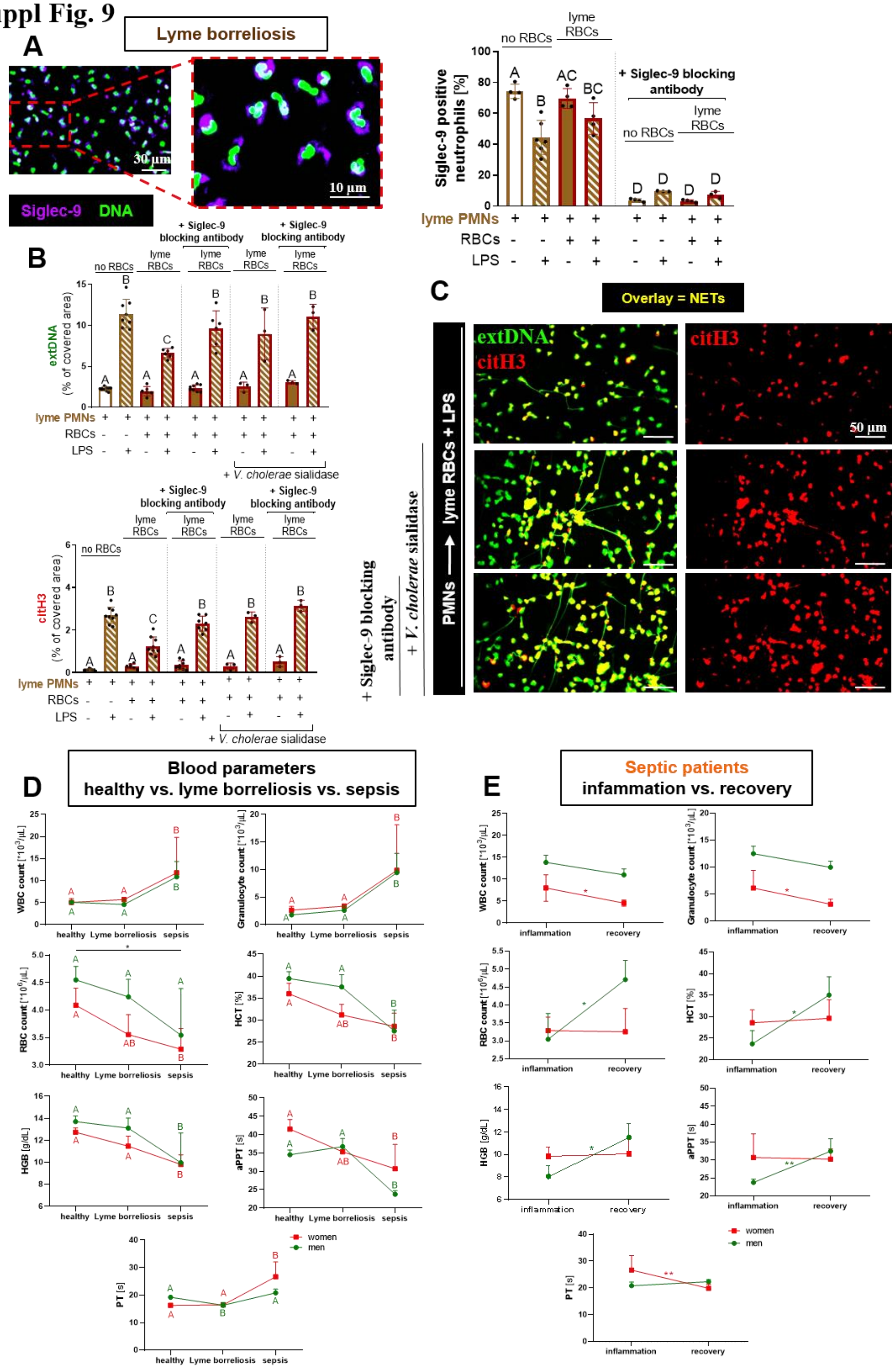
A
Lyme borreliosis
30 µm
10 µm
Siglec-9 DNA
Siglec-9 positive neutrophils [%]
no RBCs
lyme RBCs
+ Siglec-9 blocking antibody
lyme PMNs
RBCs
LPS
B
extDNA (% of covered area)
citH3 (% of covered area)
+ V. cholerae sialidase
C
Overlay = NETs
extDNA
citH3
50 µm
PMNs → lyme RBCs + LPS
+ Siglec-9 blocking antibody
+ V. cholerae sialidase
D
Blood parameters
healthy vs. lyme borreliosis vs. sepsis
WBC count [*10³/µL]
Granulocyte count [*10³/µL]
RBC count [*10⁶/µL]
HCT [%]
HGB [g/dL]
aPPT [s]
PT [s]
healthy
Lyme borreliosis
sepsis
women
men
E
Septic patients
infammation vs. recovery
inflammation
recovery

Fig. S9. Human erythrocytes inhibit NET release in patients with Lyme disease, but not sepsis, unless patients are in the recovery phase. Erythrocytes and neutrophils were isolated from whole blood of patients with Lyme disease. *Ex vivo,* the cells were cultured either as monocultures (PMN only) or in co-culture with autologous RBCs at a PMN:RBC ratio of 1:4 and subsequently incubated for 3 h in the presence of LPS (50 µg/mL). In selected experiments, monoclonal blocking antibodies against Siglec-9 (0.075 µg/mL) were added 30 min before the addition of RBCs and/or LPS. In other experimental settings, co-cultures were supplemented with RBCs pre-treated with *V. cholerae* sialidase (0.125 U, 1 h), or with a combination of both Siglec-9 blockade and sialidase treatment. (A) Surface expression of Siglec-9 (purple) was evaluated on neutrophils, as well as Siglec-9 neutralization with a blocking antibody, as shown by representative images and data quantification. (B) Quantitative data showing the percentage of area occupied by individual NET components, including extracellular DNA (extDNA) and citrullinated histone H3 (citH3), released by neutrophils, as determined by ImageJ analysis. (C) Representative images of NET formation in the presence of RBCs and LPS under the indicated experimental modifications (sialidase treatment and/or Siglec-9 receptor blockade). Visualization: extracellular DNA (extDNA, green), detected using the fluorescent DNA dye SYTOX Green; citrullinated histone H3 (citH3, red), detected using polyclonal primary anti-citH3 antibodies and Cy3-conjugated secondary antibodies; Siglec-9 (purple), detected using a monoclonal anti-Siglec-9 antibody conjugated to Alexa Fluor 647. Images were acquired at 20× and 40× optical magnification. Scale bars indicate 10, 30, or 50 µm, as labeled in the images. (D) Changes in selected peripheral blood parameters in healthy volunteers, patients with Lyme disease, and patients with sepsis, stratified by sex. Peripheral blood analyses were performed in parallel with cell isolation using an automated 3-diff hematology analyzer. (E) Sex-dependent changes in selected hematological parameters in septic patients during the early inflammatory phase (1-2 days) and the recovery phase (10-14 days). Results are presented as mean ± SD from at least three fields of view/patient. Statistically significant differences were marked with letter symbols according to one-way ANOVA (Bonferroni post hoc). Asterisks in the graphs indicate statistically significant differences according to the Student-t test ($*.01 < P \leq .05$; $**.001 < P \leq .01$); $n \geq 3$; aPPT - activated partial thromboplastin time, F – female, GRAN – granulocytes, HCT – hematocrit, HGB – hemoglobin concentration, M – male, PMN – neutrophils, PT - prothrombin time, RBC – erythrocytes, WBC – white blood cells (total leukocytes).

Suppl Fig. 10

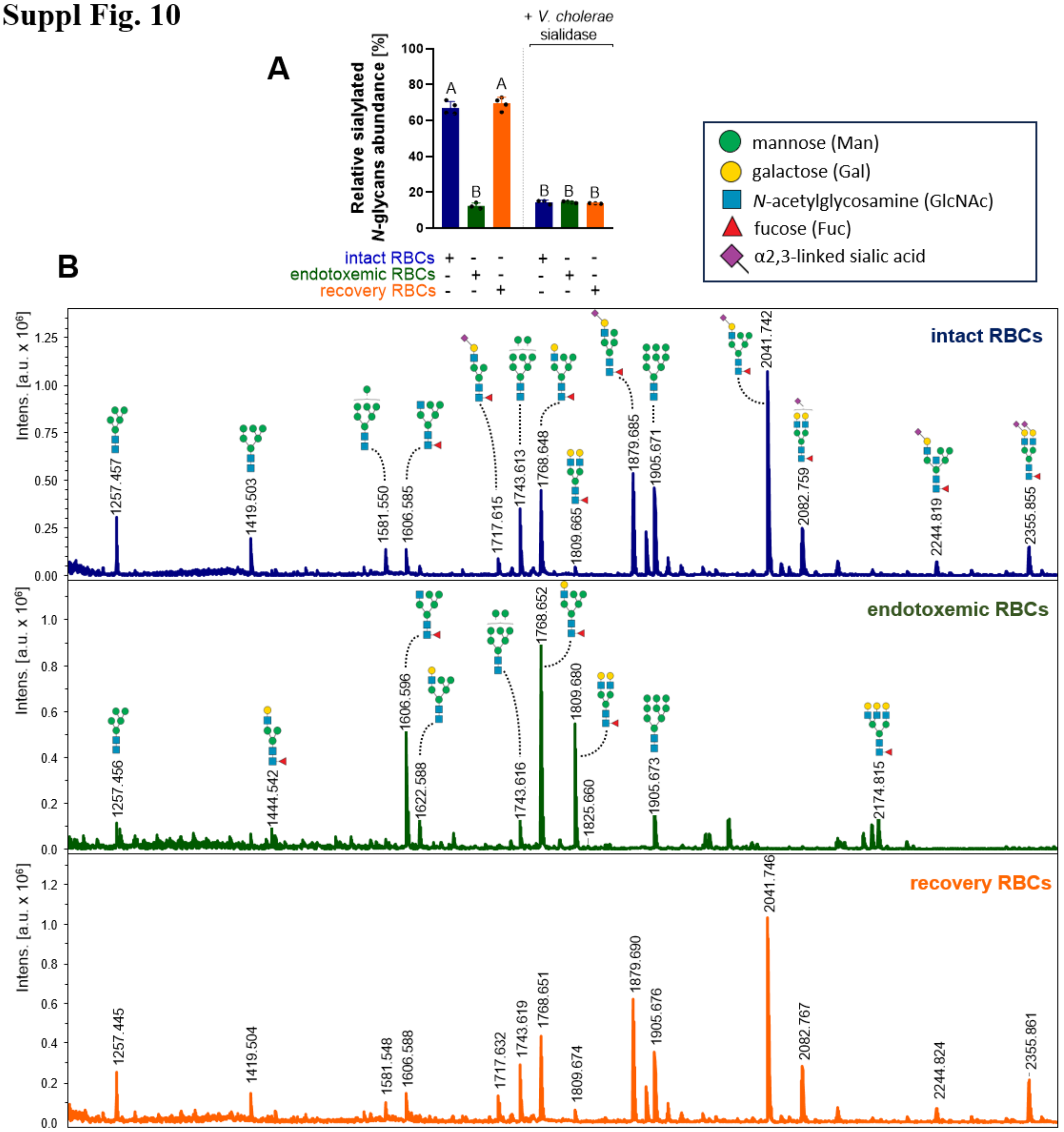

Fig. S10. During systemic inflammation, nearly complete desialylation of erythrocytes occurs. Erythrocytes were isolated from whole blood of healthy and endotoxemic C57BL/6J mice at the early phase of inflammation (8 hours; i.p. LPS, 1 mg/kg body weight). Additionally, RBCs were isolated during the recovery phase (resolution of the inflammatory response; 6 days after i.p. administration of LPS at 1 mg/kg body weight). The isolated RBCs at a concentration of 5 × 108 per sample were treated with *V. cholerae* sialidase (0.125 U for 1 hour) or left untreated, and then analyzed by MALDI-ToF mass spectrometry. (A) Changes in N-glycan profiles on the erythrocyte surface under homeostatic/inflammatory/recovery conditions. Some cells were pretreated with *V. cholerae* sialidase before evaluation. Mass spectra were acquired in the m/z range 500 – 5000, in positive-ion reflectron mode, and N-glycan profile analysis was performed using GlycoWorkbench platform. Individual peaks in the spectra were assigned their corresponding masses. (B) Representative spectra are shown. Structures of individual glycans are labelled according to the Symbol Nomenclature for Glycans (SNFG). Results are presented as mean ± SD. Statistically significant differences were marked with letter symbols according to one-way ANOVA (Bonferroni post hoc); n ≥ 3; RBC – erythrocytes.

Suppl Fig. 11

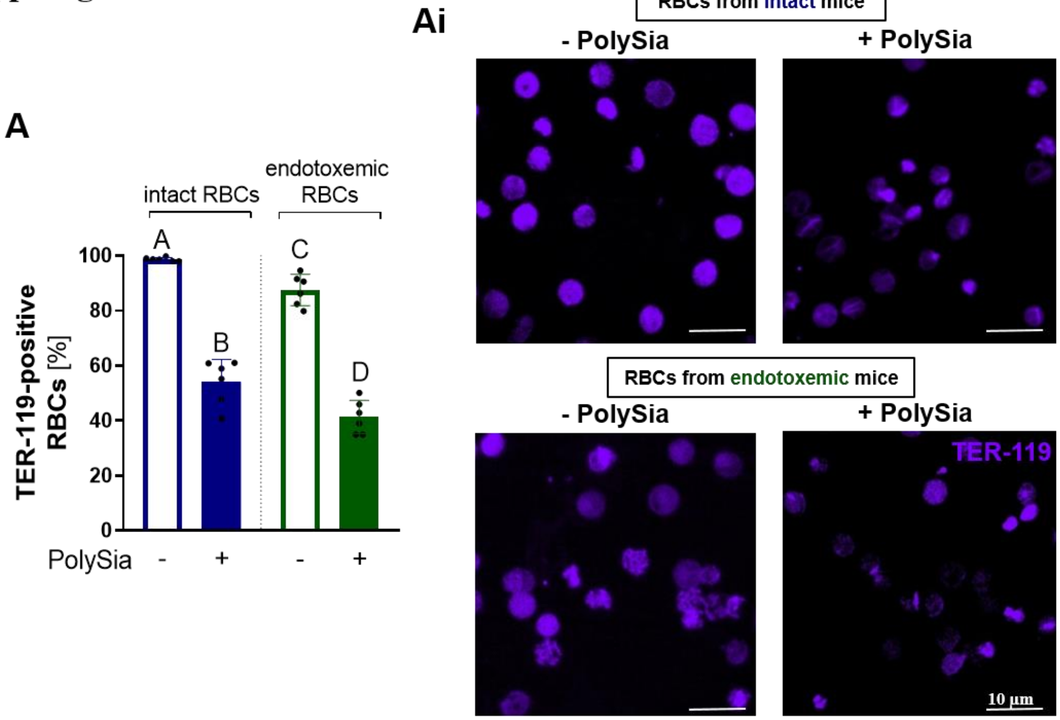

Fig. S11. Polysialic acid (PolySia) reduces TER-119 staining in both intact and endotoxemic erythrocytes, indirectly indicating its attachment to the erythrocyte surface. (A-Ai) Erythrocytes were isolated from the whole blood of intact or endotoxemic (8 h) C57BL/6J mice. Isolated cells were treated with exogenous polysialic acid (PolySia, 10 µg/ml) for 6 h. Subsequently, cells were stained with monoclonal antibodies against TER-119 (purple). (A) The percentage of TER-119-positive RBCs was analyzed with ImageJ and expressed as % of cells per cell number. (Ai) Representative confocal images showing changes in TER-119 staining in intact and endotoxemic RBCs with PolySia addition. Images were acquired at 40× optical magnification; scale bars indicate 10 µm. Results are presented as mean ± SD from at least three fields of view. Statistically significant differences were marked with letter symbols according to one-way ANOVA (Bonferroni post hoc); n ≥ 3; RBC – erythrocytes.

Suppl Fig. 12

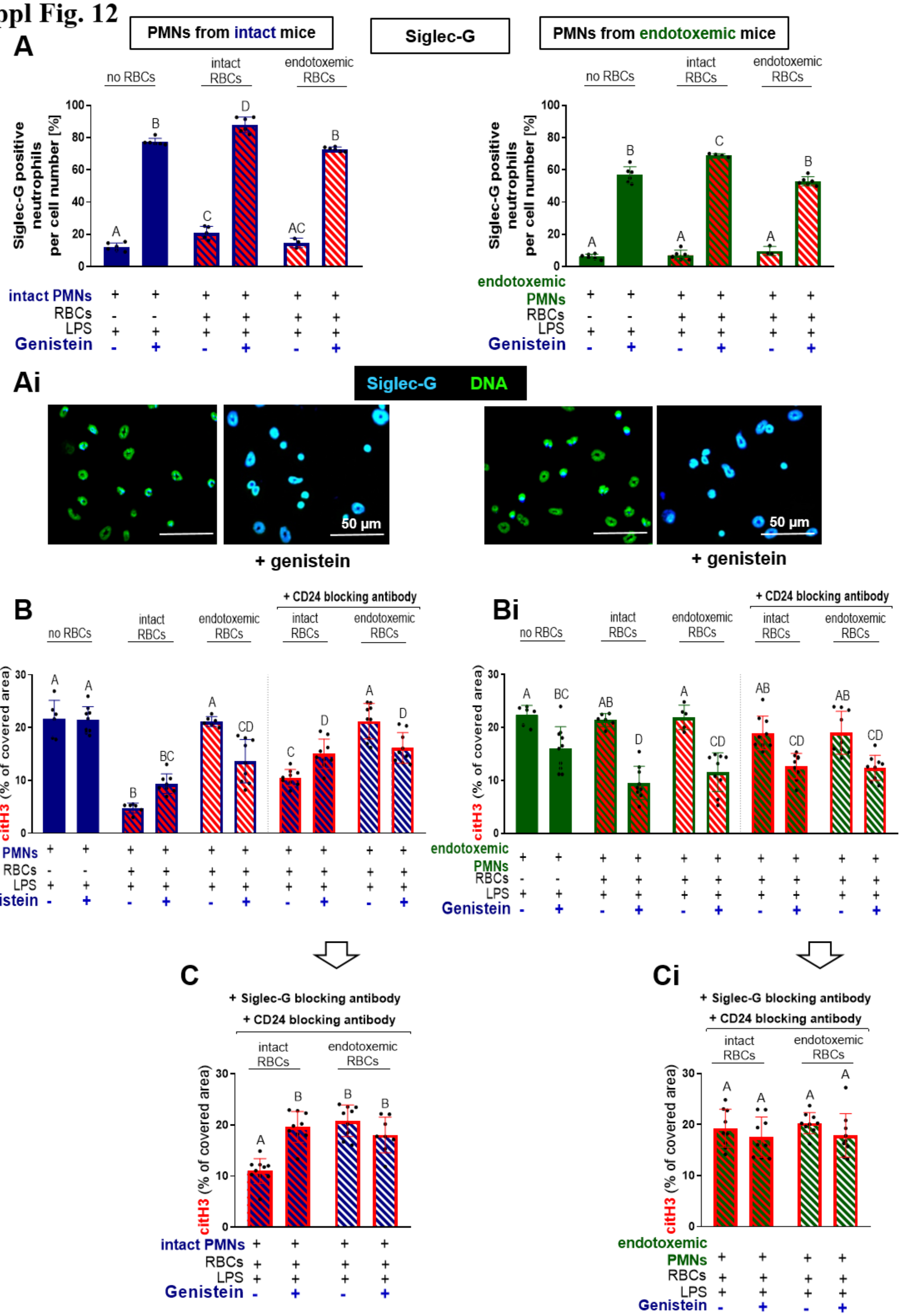
A
PMNs from intact mice
Siglec-G
PMNs from endotoxemic mice
no RBCs
intact RBCs
endotoxemic RBCs
Siglec-G positive neutrophils per cell number [%]
intact PMNs
endotoxemic PMNs
RBCs
LPS
Genistein
Ai
Siglec-G DNA
50 µm
+ genistein
B
Bi
+ CD24 blocking antibody
citH3 (% of covered area)
C
Ci
+ Siglec-G blocking antibody
+ CD24 blocking antibody

Fig. S12. Genistein markedly increases Siglec-G expression, thereby restoring NET inhibition by endotoxemic erythrocytes. Erythrocytes and neutrophils were isolated from whole blood and bone marrow of healthy and endotoxemic (8 hours; i.p. LPS, 1 mg/kg body weight) C57BL/6J mice. The cells were cultured with or without mixing (e.g., healthy PMNs with endotoxemic RBCs) and then stimulated with LPS (50 µg/ml) for 6 hrs. (A) Surface expression of Siglec-G was evaluated on neutrophils isolated from mice pre-treated with genistein, preserving its surface expression (i.p., 150 mg/kg body weight) 8 hrs before endotoxemia induction or without it: (left) neutrophils isolated from healthy versus (right) endotoxemic mice were compared when cultured with RBC from either intact or inflamed mice. (Ai) Representative images of the Siglec-G signal (blue) on neutrophils of healthy (intact) and endotoxemic mice. Cell nuclei were counterstained with SYTOX green. Merged images (Siglec-G + DNA) were acquired at 40× optical magnification; scale bar indicates 50 µm. (B-C) Under the same conditions as in (A), the NET formation was evaluated by application of antibodies detecting citrullinated histone 3 (citH3). Additionally, some RBCs were pretreated with CD24-blocking antibodies (5 µg/ml) for 1 h before co-culture with neutrophils. (B, C) Intact or endotoxemic neutrophils were compared when cultured with RBCs from either intact or endotoxemic mice. (C, Ci) The double blocking was applied as both Siglec-G and CD24 were neutralized with appropriate antibodies. Results are presented as mean ± SD from at least three fields of view. Statistically significant differences were marked with letter symbols according to one-way ANOVA (Bonferroni post hoc); n ≥ 3; PMN – neutrophils, RBC – erythrocytes.

Tables

Table S1. Characteristics of healthy volunteers, patients with Lyme disease, and sepsis (in the early and late phases), donating blood cells. Peripheral blood analyses were performed in parallel with the cell isolation procedure using a 3-diff automatic hematology analyzer. The results for individual parameters, stratified by group and sex, are expressed as the mean ± SD. RBC – red blood cells (total count), WBC – white blood cells (total leukocytes count), GRAN –granulocytes, HCT – hematocrit, HGB – hemoglobin concentration, F – female, M – male.

GROUP /total numer of individuals/	SEX /number/	AGE /average in years/	AGE /range in years/	RBC [$*10^6/\mu L$]	WBC [$*10^3/\mu L$]	GRAN [$*10^3/\mu L$]	HCT [%]	HGB [g/dL]
**Healthy Volunteers** n=5	F, n=3	55	28 – 85	4,1 ± 0,3	5,5 ± 0,9	2,6 ± 0,7	36 ± 2,4	12,7± 0,4
	M, n=2	61,5	35 - 88	4,6 ± 0,2	5,1 ± 0,4	1,8 ± 0,2	39,5 ± 1,6	13,7± 0,5
**Septic patients** (early phase; 1-2 days post diagnosis) n=5	F, n=3	58	37-78	3,3 ± 0,4	11,8 ± 8,1	9,9 ± 8,3	28,6 ± 3	9,8 ± 0,9
	M, n=2	54	37-71	3,5 ± 0,8	10,8 ± 3,5	9,5 ± 3,5	27,6 ± 4,7	10 ± 2,7
**Septic patients – recovery phase** (late phase; 10-14 days post diagnosis) n=5	F, n=3	58	37-78	3,3 ± 0,6	5,1 ± 1,2	3.1 ± 0,9	29,7 ± 4,3	10,1 ± 1,5
	M, n=2	54	37-71	4,7 ± 0,5	5.9 ± 0,6	10 ± 1,2	35 ± 4,3	11,5 ± 1,2
**Patients with Lyme disease** n=5	F, n=1	61	61	3,6 ± 0,4	5,7 ± 0,4	3,4 ± 0,4	31,2 ± 2,4	11,5 ± 0,9
	M, n=4	69,5	57-78	4,24 ± 0,3	4,6 ± 1,1	2,6 ± 0,8	37,6 ± 2,8	13,1 ± 0,9

Dataset S1 (separate file). Characterization of N-glycans derived from intact, endotoxemic, and recovery murine erythrocytes (RBCs). N-glycans were analyzed individually, as well as in the form of derived glycan traits (sialylation), by positive-ion reflection mode MALDI-ToF-MS. For structural assignment, m/z values together with common knowledge of glycobiology were used. The average ± SD of the relative percentage intensity of each peak is given in the table. The symbols used for oligosaccharides are: Hex, hexose; HexNAc, N-acetylhexosamine; dHex, deoxyhexose; L, E, α2,3-linked sialic acid; ND - not detected, RBC - erythrocytes.

SI References

1. I. Cichon, W. Ortmann, E. Kolaczkowska, Metabolic pathways involved in formation of spontaneous and lipopolysaccharide-induced neutrophil extracellular traps (NETs) differ in obesity and systemic inflammation. *Int. J. Mol. Sci.* **22**, 7718 (2021).
2. G. M. Kiser, Z. M., Lizcano, A., Nguyen, J., Becker, G. L., Belcher, J. D., Varki, A. P., & Vercellotti, Decreased erythrocyte binding of Siglec-9 increases neutrophil activation in sickle cell disease. *Blood Cells. Mol. Dis.* **81**, 102399 (2020).
3. K. R. Reiding, D. Blank, D. M. Kuijper, A. M. Deelder, M. Wuhrer, High-Throughput Profiling of Protein N-Glycosylation by MALDI-TOF-MS Employing Linkage-Specific Sialic Acid Esterification. *Anal. Chem.* **86**, 5784–5793 (2014).
4. A. Ceroni, *et al.*, GlycoWorkbench: A tool for the computer-assisted annotation of mass spectra of glycans. *J. Proteome Res.* **7**, 1650–1659 (2008).
