# Supplementary material for: Loss of erythrocyte sialic acid in sepsis disrupts inhibitory Siglec interactions, driving neutrophil hyperactivation and NET outspread
Source: Proc Natl Acad Sci U S A. 2026 Jun 10;123(24):e2536989123. doi: 10.1073/pnas.2536989123 (PMC13273359; doi:10.1073/pnas.2536989123)
Supplement: Supplementary file 2 — Dataset S01 (PDF) [file pnas.2536989123.sd01.pdf]

Dataset S1. Characterization of N-glycans derived from intact, endotoxemic and recovery murine erythrocytes (RBCs). *N*-glycans were analysed individually, as well as in the form of derived glycan traits (sialylation), by positive-ion reflection mode MALDI-ToF-MS. For structural assignment *m/z* values together with common knowledge of glycobiology were used. The average ± SD of relative percentage intensity of each peak was given in the table.The symbols used for oligosaccharides are: Hex, hexose; HexNAc, N-acetylhexosamine; dHex, deoxyhexose; L, E, α2,3-linked sialic acid; ND - not detected, RBC - erythrocytes.

		INTACT RBCs			RECOVERY RBCs		
**Composition**	**Theoretical m/z**	Observed m/z (average)	**average abundance [%]**	SD	Observed m/z (average)	**average abundance [%]**	SD
Hex5HexNAc2	**1257,42**	1257,44	3,09	0,40	1257,44	2,78	0,16
Hex6HexNAc2	**1419,48**	1419,49	2,11	0,20	1419,58	1,95	0,78
Hex7HexNAc2	**1581,53**	1581,54	1,98	0,23	1581,55	1,50	0,29
Hex5HexNAc3dHex1	**1606,56**	1606,57	2,34	0,25	1606,58	2,12	0,31
Hex4HexNAc3L1dHex1	**1717,59**	1717,60	2,01	0,58	1717,62	2,10	0,32
Hex8HexNAc2	**1743,58**	1743,60	4,81	1,02	1743,61	4,71	0,96
Hex6HexNAc3dHex1	**1768,61**	1768,63	7,85	1,06	1768,64	7,59	1,02
Hex5HexNAc4dHex1	**1809,64**	1809,65	1,08	0,19	1809,67	1,14	0,15
Hex5HexNAc3L1dHex1	**1879,64**	1879,66	12,38	1,13	1879,68	12,04	0,44
Hex6HexNAc3L1	**1895,64**	1895,66	4,18	0,49	1895,67	4,14	0,32
Hex9HexNAc2	**1905,63**	1905,65	7,30	1,73	1905,67	6,79	1,62
Hex6HexNAc3L1dHex1	**2041,70**	2041,72	24,94	1,37	2041,74	24,01	0,62
Hex5HexNAc4L1dHex1	**2082,72**	2082,74	6,84	0,51	2082,76	6,77	0,45
Hex6HexNAc4L1dHex1	**2244,78**	2244,80	2,00	0,10	2244,82	2,30	0,18
Hex5HexNAc4L2dHex1	**2355,81**	2355,83	5,99	1,14	2355,85	6,64	0,68
Hex6HexNAc4L2	**2371,80**	2371,82	0,73	0,50	2371,84	1,17	0,25
Hex7HexNAc4L1dHex1	**2406,83**	2406,85	1,27	0,17	2406,86	1,35	0,08
Hex6HexNAc5L1dHex1	**2447,86**	2447,86	0,81	0,10	2447,88	0,89	0,05
Hex6HexNAc6dHex2	**2523,91**	2523,87	2,39	0,80	2523,89	1,98	0,38
Hex6HexNAc5L2dHex1	**2720,94**	2720,96	1,36	0,22	2720,98	1,70	0,31
Hex6HexNAc5L3dHex1	**2994,02**	2994,05	1,14	0,30	2994,06	1,46	0,32
E1Hex8HexNAc7	**3078,11**	3078,07	1,28	0,50	3078,07	1,70	0,47
Hex7HexNAc7L1dHex2	**3162,13**	3162,09	0,74	0,59	3162,10	1,20	0,37
E1Hex9HexNAc8	**3443,24**	3443,22	1,36	0,35	3443,21	1,98	0,62

		ENDOTOXEMIC RBCs	
**Composition**	**Theoretical m/z**	Observed m/z (average)	**average abundance [%]**
Hex5HexNAc2	**1257,42**	1257,47	2,02
Hex4HexNAc3dHex1	**1444,51**	1444,56	1,72
Hex5HexNAc3dHex1	**1606,56**	1606,62	12,00
Hex6HexNAc3	**1622,55**	1622,61	2,97
Hex5HexNAc4	**1663,58**	1663,63	1,55
Hex8HexNAc2	**1743,58**	1743,64	3,45
Hex6HexNAc3dHex1	**1768,61**	1768,68	24,26
Hex7HexNAc3	**1784,61**	ND	ND
Hex5HexNAc4dHex1	**1809,64**	1809,71	15,19
Hex6HexNAc4	**1825,63**	1825,70	0,19
Hex9HexNAc2	**1905,63**	1905,70	5,01
Hex6HexNAc4dHex1	**1971,69**	1971,76	2,27
Hex7HexNAc4dHex1	**2133,74**	2133,81	1,26
Hex6HexNAc5dHex1	**2174,77**	2174,85	5,33
Hex6HexNAc6dHex2	**2523,91**	2523,94	1,17
Hex7HexNAc6dHex1	**2539,90**	2539,98	3,04
Hex7HexNAc7dHex2	**2889,04**	2889,06	1,29
E1Hex5HexNAc5L1dHex3	**2897,05**	2897,09	2,68
Hex8HexNAc7dHex1	**2905,04**	2905,12	2,25
Hex6HexNAc7L3	**3254,13**	3254,21	1,52
E1Hex6HexNAc6L1dHex3	**3262,18**	3262,24	2,61
Hex9HexNAc8dHex1	**3270,17**	3270,27	2,55
Hex7HexNAc8L3	**3619,26**	3619,36	1,48
E1Hex7HexNAc7L1dHex3	**3627,31**	3627,39	2,71
Hex6HexNAc6L3dHex4	**3635,28**	3635,41	1,46
